# Spike Pattern Structure Influences Synaptic Efficacy Variability under STDP and Synaptic Homeostasis. II: Spike Shuffling Methods on LIF Networks

**DOI:** 10.3389/fncom.2016.00083

**Published:** 2016-08-09

**Authors:** Zedong Bi, Changsong Zhou

**Affiliations:** ^1^State Key Laboratory of Theoretical Physics, Institute of Theoretical Physics, Chinese Academy of SciencesBeijing, China; ^2^Department of Physics, Hong Kong Baptist UniversityKowloon Tong, Hong Kong; ^3^Centre for Nonlinear Studies, Beijing-Hong Kong-Singapore Joint Centre for Nonlinear and Complex Systems, Institute of Computational and Theoretical Studies, Hong Kong Baptist UniversityKowloon Tong, Hong Kong; ^4^Beijing Computational Science Research CenterBeijing, China; ^5^Research Centre, Hong Kong Baptist University Institute of Research and Continuing EducationShenzhen, China

**Keywords:** spike pattern structure, synaptic plasticity, efficacy variability, STDP, synaptic homeostasis, spike shuffling methods

## Abstract

Synapses may undergo variable changes during plasticity because of the variability of spike patterns such as temporal stochasticity and spatial randomness. Here, we call the variability of synaptic weight changes during plasticity to be *efficacy variability*. In this paper, we investigate how four aspects of spike pattern statistics (i.e., synchronous firing, burstiness/regularity, heterogeneity of rates and heterogeneity of cross-correlations) influence the efficacy variability under pair-wise additive spike-timing dependent plasticity (STDP) and synaptic homeostasis (the mean strength of plastic synapses into a neuron is bounded), by implementing spike shuffling methods onto spike patterns self-organized by a network of excitatory and inhibitory leaky integrate-and-fire (LIF) neurons. With the increase of the decay time scale of the inhibitory synaptic currents, the LIF network undergoes a transition from asynchronous state to weak synchronous state and then to synchronous bursting state. We first shuffle these spike patterns using a variety of methods, each designed to evidently change a specific pattern statistics; and then investigate the change of efficacy variability of the synapses under STDP and synaptic homeostasis, when the neurons in the network fire according to the spike patterns before and after being treated by a shuffling method. In this way, we can understand how the change of pattern statistics may cause the change of efficacy variability. Our results are consistent with those of our previous study which implements spike-generating models on converging motifs. We also find that burstiness/regularity is important to determine the efficacy variability under asynchronous states, while heterogeneity of cross-correlations is the main factor to cause efficacy variability when the network moves into synchronous bursting states (the states observed in epilepsy).

## 1. Introduction

Variability is a prominent feature of the neuronal activities. The neurons in the same population may respond quite differently to the same stimulus (structural variability), and the responses of a neuron to the same stimulus can also differ in different trials (trial variability). Structural variability comes from the heterogeneity of neuronal responsive properties and the randomness of inter-neuronal connections. It is found that even the same type of neurons may have different responsive properties due to the difference in the gene expression of membrane ion channels (Schulz et al., 2006; Padmanabhan and Urban, 2010); and the strengths of synapses may span several magnitudes (Song et al., 2005; Buzsáki and Mizuseki, 2014), continuously changing with time (Zucker and Regehr, 2002; Keck et al., 2008). Trial variability partly comes from biomolecular noises such as the open and close of ion channels and the release of synaptic vesicles (see Faisal et al., 2008 for review). Such noises may enter any stage of information processing in the brain, from perception and decision making to motion generation (Faisal et al., 2008), influencing the reliability and timing of action potentials (Allen and Stevens, 1994; Zador, 1998; Dorval and White, 2005; Faisal and Laughlin, 2007), especially in neurons with thin axons (Faisal et al., 2005). Additionally, a neuronal network may have internal states such as slow synaptic currents, the strengths of the synapses under short-term plasticity, and the phase of the internal oscillation (Mongillo et al., 2008; Buonomano and Maass, 2009; VanRullen et al., 2011); and its response may vary depending on these internal states (Cohn et al., 2015; Daie et al., 2015), combined with being modulated or gated by inputs from the other brain areas (Masquelier, 2013; Lin et al., 2015). If the dynamics of the network exhibits deterministic chaos, such as theoretically suggested for the networks under excitatory-inhibitory balanced state (van Vreeswijk and Sompolinsky, 1998; Monteforte and Wolf, 2010; Ostojic, 2014), then the responsive variability will be exacerbated due to the high sensitivity to noises and initial conditions.

The nervous system is able to adapt its response to external stimuli by changing the strengths of its synapses. As the synaptic changes depend on the spike timings of the pre- and post-synaptic neurons (Dan and Poo, 2006; Caporale and Dan, 2008; Markram et al., 2012), the variability of neuronal activities must result in variability of synaptic changes. Besides, synaptic plasticity is also influenced by other factors such as neuromodulators (Bailey et al., 2000; Berke and Hyman, 2000) and series of pre- and post-synaptic biomolecular mechanisms (Graupner and Brunel, 2010; Yang and Calakos, 2013), and variability may come into any of these factors. Overall, synaptic plasticity is a “noisy” process, and we call the variability of the synaptic changes during plasticity induced by the stochasticity and randomness of neuronal networks to be the *efficacy variability*. See Section 2.1 for more discussions on the definition of efficacy variability.

**Figure 1:**
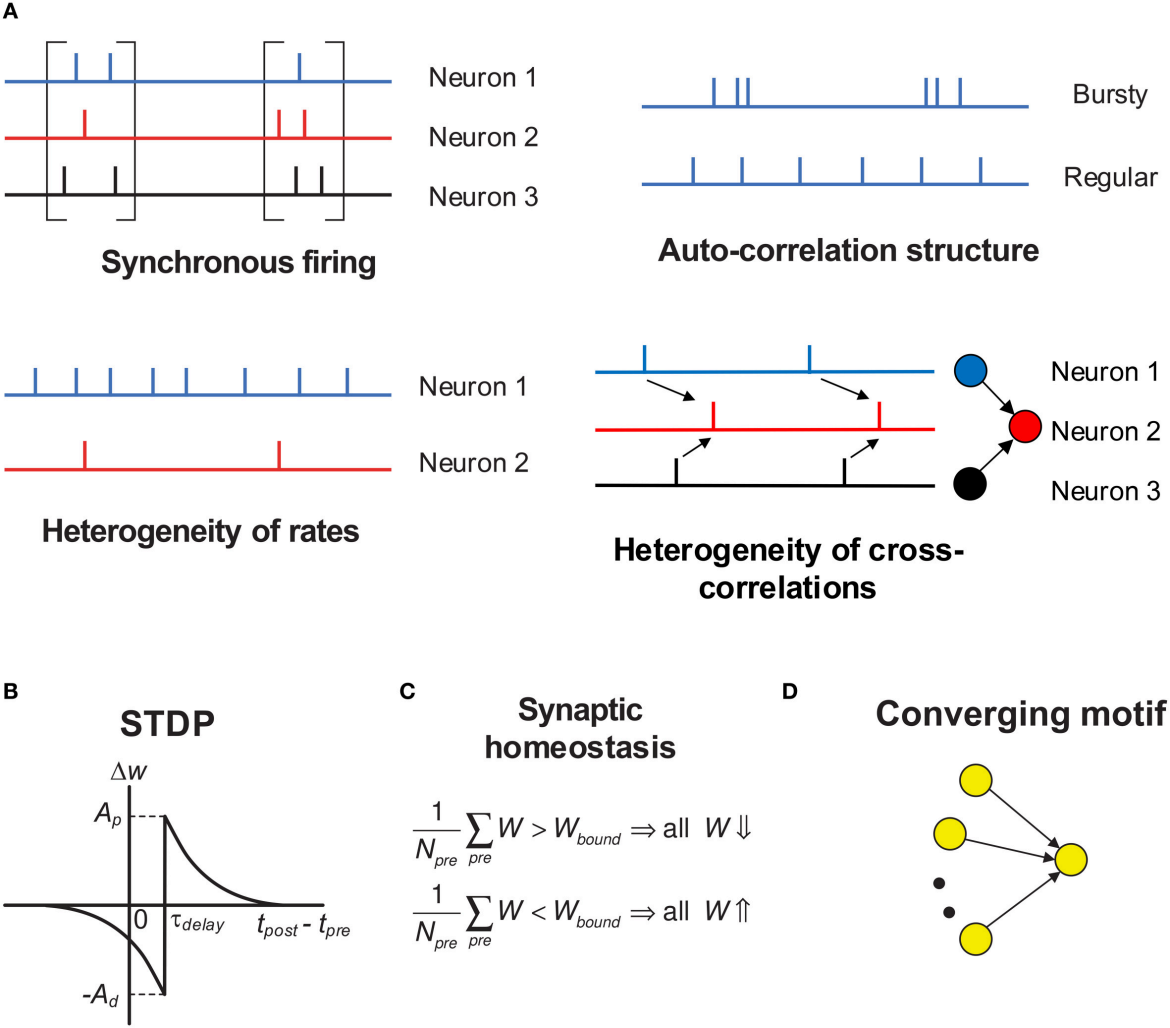
**Schematic of the key concepts in our modeling work**. **(A)** The four aspects of pattern structure studied in this paper. “Synchronous firing” typically means the spurt of firing activity of a population; in this paper, it also represents the time fluctuation of the population rate in asynchronous spike patterns. For asynchronous spike patterns, “auto-correlation structure” reflects the burstiness/regularity of the spike trains, which is quantified by coefficient of variance (*CV*) in this paper. Here, by “burstiness,” we typically mean the irregular structure of spike trains, instead of the regular burstiness in the spike patterns of, say, central pattern generator. For spike trains in synchronous states, we consider three types of “auto-correlation structure” to reflect the burstiness/regularity features of the spike patterns (see **Figure 4**). By “heterogeneity of rates,” we mean that the time-averaged firing rates are different for different neurons. By “heterogeneity of cross-correlations,” we mean that different pre-synaptic neurons of a neuron tends to fire spikes at different times relative to the spikes of the neuron. For example, in the right-bottom subplot, before a spike of neuron 2, neuron 1 tends to fire before neuron 3. **(B)** The STDP time window used in our work. Note that the axons in our work have time delay τ_*delay*_, and the synapses are updated according to the spike time of the post-synaptic neuron and the time that the pre-synaptic spike arrives at the terminal. The STDP updatings of all spike pairs are summed together. **(C)** Synaptic homeostasis. The synapses input to a neuron are subject to a bound on their mean strength: when their mean strength is different from this bound, all the incoming synapses of that neuron will undergo an adjustment. **(D)** Converging motif, on which we conducted all the simulations in the previous paper (Bi and Zhou, 2016). Modeling details are presented in Section 2.

In this paper, we focus on discussing the influence of variability of neuronal activities on efficacy variability, in the context of spike-timing dependent plasticity (STDP) (Gerstner et al., 1996; Dan and Poo, 2006; Caporale and Dan, 2008; Markram et al., 2012). Under STDP, synaptic plasticity is driven by spike trains, so the efficacy variability is caused by the temporal stochasticity and spatial randomness of spike trains. Additionally, spike trains may exhibit a variety of statistical features, which form rich spike pattern structures. Groups of neurons may spurt firing activity (*synchronous firing*) (Kamioka et al., 1996; Buzsáki and Draguhn, 2004; Bartos et al., 2007), the spike train of a single neuron can be bursty or regular (*auto-correlation structure*) (Softky and Koch, 1993; Schwindt and Crill, 1999; Jacob et al., 2012), firing rates of cortical neurons are typically heavily-skewed distributed *in vivo* (*heterogeneity of rates*) (Shafi et al., 2007; O'Connor et al., 2010; Buzsáki and Mizuseki, 2014), and the spike trains of different neurons also display rich inter-dependences (*heterogeneity of cross-correlations*) (Funahashi and Inoue, 2000; Schneidman et al., 2006; Ostojic et al., 2009; Trousdale et al., 2012). See Figure 1A for the concepts above. Under STDP, synaptic plasticity is induced by spike trains, so these spike pattern structures must have strong influence on efficacy variability, inducing neuronal networks with sharply different structures even under the same population rate. How these spike pattern structures may influence the efficacy variability is the topic we are interested in.

**Figure 2:**
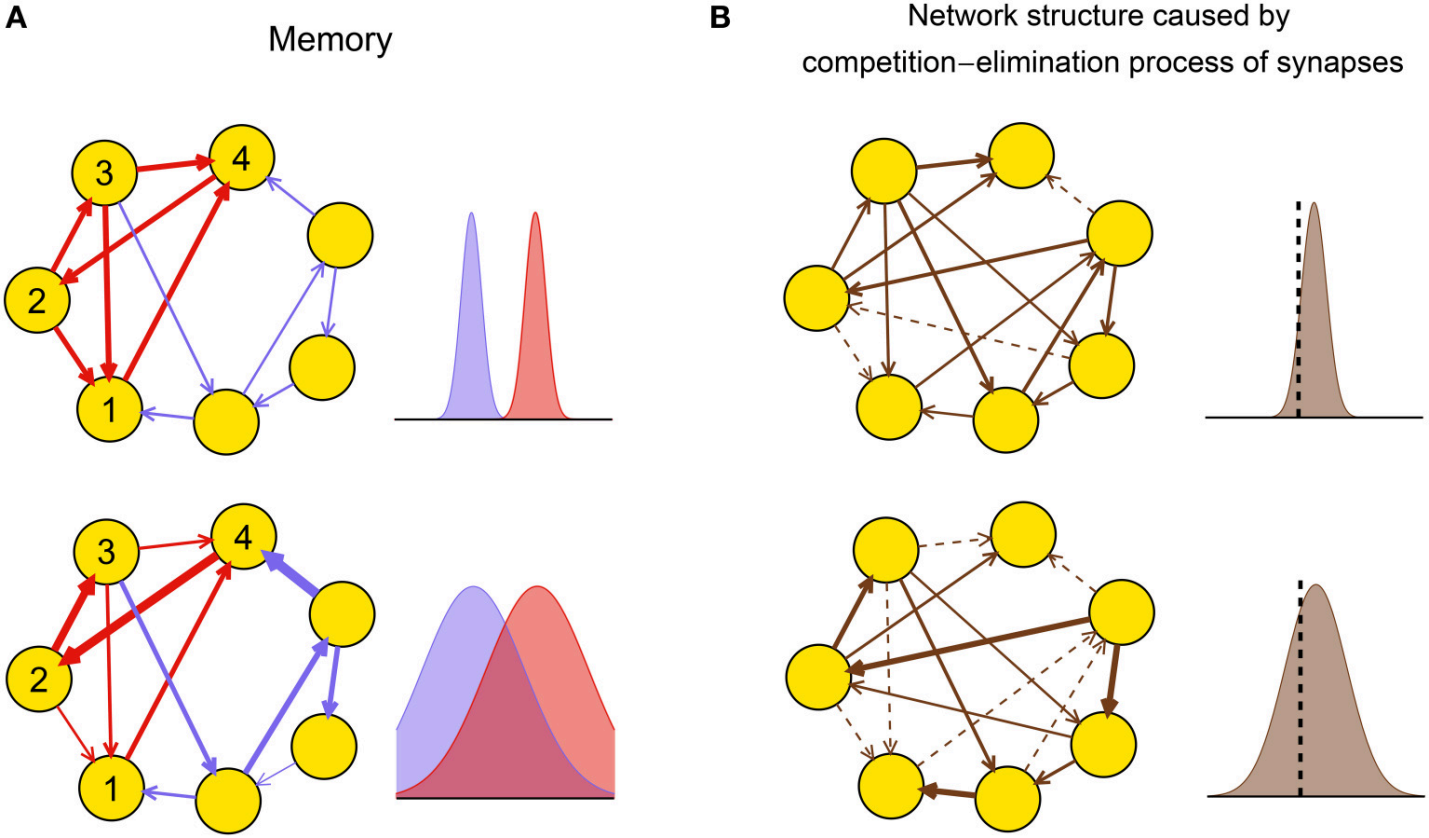
**Biological implications of efficacy variability**. **(A)** A network of excitatory neurons stores a memory using its attractor dynamics after the intra-connections within a sub-population (here, neurons 1–4) are strengthened (the inhibitory population that keeps the total activity of the network is not shown). When the efficacy variability is small (upper row), this subpopulation will exhibit persistently high activity if a sufficiently large number of neurons in the subpopulation have high activities initially, so the memory is retrieved. When the efficacy variability is large (lower row), this memory retrieval will fail even if the mean strength of the intra-connections (red) is stronger than that of the other ones (blue). The widths of arrows represent synaptic strengths. The subplots on the right represent the weight distributions of the blue and red synapses shown in the subplots on the left. **(B)** Efficacy variability causes different network structures by controlling the degree of synaptic competition. When the efficacy variability is small (upper), only a few synapses are weaker than the elimination threshold (black dashed vertical line) and get eliminated during neural development, so most synapses are left and their strengths tend to be uniform; when the efficacy variability is large (lower), more synapses are eliminated, and the left ones are more heterogeneous and also stronger than the upper case on average. Dashed arrows represent eliminated synapses. The subplots on the right represent the weight distributions of the synapses before the elimination process.

Theoretical and experimental results suggest that the dynamic pattern of a neuronal network during plasticity may strongly influence the functional performance of the resulting network after plasticity by influencing the efficacy variability. For example, large efficacy variability may blur the connection patterns that are required to successfully embed memory into the network (Figure 2A), thereby destroying memory. Experimentally, it is found that gamma oscillations are important for memory formation under normal physiological conditions (Sederberg et al., 2007; Jutras et al., 2009; Yamamoto et al., 2014); but too strong synchrony, such as that in epilepsy (Gulyás and Freund, 2015), is instead detrimental to memory (Butler and Zeman, 2008). These observations suggest that efficacy variability may get its smallest value under weak synchrony, and get larger at both asynchronous and strong synchronous states. As another example, the efficacy variability may influence the competition-and-elimination process of the synapses under development (Cancedda and Poo, 2009), resulting in networks with different sparsities and synaptic strengths (Figure 2B). Experimentally, it is found that if the spontaneous activity of the medial nucleus of the trapezoid body (MNTB) in the auditory pathway during early development is modified using genetic methods, then its feedforward projection to the lateral superior olive (LSO) becomes denser and weaker, which hinders the refinement of receptive fields of the LSO neurons, thereby destroying the hearing of the animal (Clause et al., 2014). This suggests that the genetically modified spontaneous activity in Clause et al. (2014) induces smaller efficacy variability than the normal one.

In our previous paper (Bi and Zhou, 2016), we studied how the four aspects of spike pattern structure shown in Figure 1A influence the efficacy variability under a conventional pair-wise additive STDP (Figure 1B) using converging motifs (Figure 1D). Additionally, we also added synaptic homeostasis which conserves the mean strength of the synapses input to a neuron (Figure 1C), which may be physiologically used to maintain the activity level in a plastic network (Turrigiano and Nelson, 2004; Turrigiano, 2011). In that paper, we first generated spike patterns using statistical models with tunable parameters, and then investigated how the efficacy variability of the converging motifs would change if the neurons fire according to the generated spike patterns with different statistics. We separated the efficacy variability (TotalV, short for “total variance”) into two parts:

(1) $$\begin{array}{r} \text{TotalV}=\text{DriftV}+\text{DiffV}, \end{array}$$

with DriftV (short for “drift variance”) being the drift part induced by the heterogeneity of change rates of different synapses caused by the spatial heterogeneity of spike trains (mainly related to heterogeneity of rates and heterogeneity of cross-correlations shown in Figure 1A), and DiffV (short for “diffusion variance”) being the diffusion part induced by the weight diffusion caused by stochasticity of spike trains (mainly related to synchronous firing and auto-correlation structure shown in Figure 1A). Our main conclusions are that (1) synchronous firing generally increases DiffV, except for spike-to-spike synchrony with good temporal precision, (2) burstiness of auto-correlation structure tends to increase DiffV, (3) heterogeneity of rates induces DriftV when potentiation and depression in STDP are not balanced, and (4) heterogeneity of cross-correlations induces DriftV together with heterogeneity of rates.

However, the research strategy of our previous paper has its limitations. For example, the spike patterns were generated by statistical models, which prevents us from understanding the contributions of different pattern statistics to the efficacy variability under biologically more plausible spike patterns. Additionally, in practice, people may want to know how the spike trains experimentally observed may influence the efficacy variability of a local neural circuit, thereby understanding the functional meanings of the statistical features of the spike trains during learning or neural development. Therefore, it is desirable to develop an approach to manipulate the spike pattern statistics in the recorded spike patterns. In this paper, we propose to use spike shuffling methods to solve this problem.

Spike shuffling methods are commonly used experimental techniques to destroy inter-spike, inter-neuron or inter-trial dependencies of spike patterns, thereby establishing significance of dependencies. It has been used when, for example, studying functional interactions between neuronal population (Narayanan and Laubach, 2009), investigating replay of spike sequence (Nádasdy et al., 1999; Ji and Wilson, 2007), identifying spatio-temporal correlations on the background of noises (Amarasingham et al., 2012), and discovering information-containing spatio-temporal correlations in neural codes (Panzeri et al., 2002; Nirenberg and Latham, 2003; Ganmora et al., 2011). For example, in Ji and Wilson (2007), to validate the replay of hippocampal and cortical neuronal activities during sleep to the pre-sleep activities, the authors compared the pre-sleep neuronal firing sequences to those during sleep as well as to the shuffled during-sleep sequences in which the neuronal firing orders are randomized. They found that the pre-sleep sequences have a higher similarity with the original during-sleep sequences than with the shuffled ones. As another example, in practice, correlations between neurons may come either from the external stimuli or from the inter-neuronal connections. In Ganmora et al. (2011), the authors assessed these two contributions by comparing the inter-neuronal correlations in the original spike pattern and in the spike pattern after trial-shuffling: here, trial-shuffling means that the authors repeated the stimulus for several trials, and different neurons in the shuffled pattern fired according to the spike trains in different trials. They found that inter-neuronal connections contribute significantly to the high-order neuronal correlations.

Spike shuffling methods may provide a good opportunity for understanding the influence of spike pattern statistics to synaptic plasticity under experimental conditions. For example, people can treat the recorded spike patterns using a spike shuffling method, to evidently change a specific pattern statistical feature while keeping the others largely intact, and then get understanding on the impact of the statistical feature on plasticity by comparing the synaptic changes after optically stimulating the neuronal population according to the spike patterns before and after shuffling. For pre-synaptic neurons, people may straightforwardly stimulate their axons. For post-synaptic neurons, to observe the synaptic changes caused by the post-synaptic neuronal activities controlled by optical stimulations instead of evoked by synaptic couplings, people may have to inject perisomatic shunting inhibition while at the same time stimulate the dendritic arbors to mimick the back-propagated action potentials caused by the imaginary firing of the post-synaptic neurons. Recent progress on the spatio-temporal precision of optogenetics prospects the feasibility of this operation simultaneously onto a number of neurons at present time or in the near future (see e.g., Fenno et al., 2011; Peron and Svoboda, 2011; Hochbaum et al., 2014).

**Figure 3:**
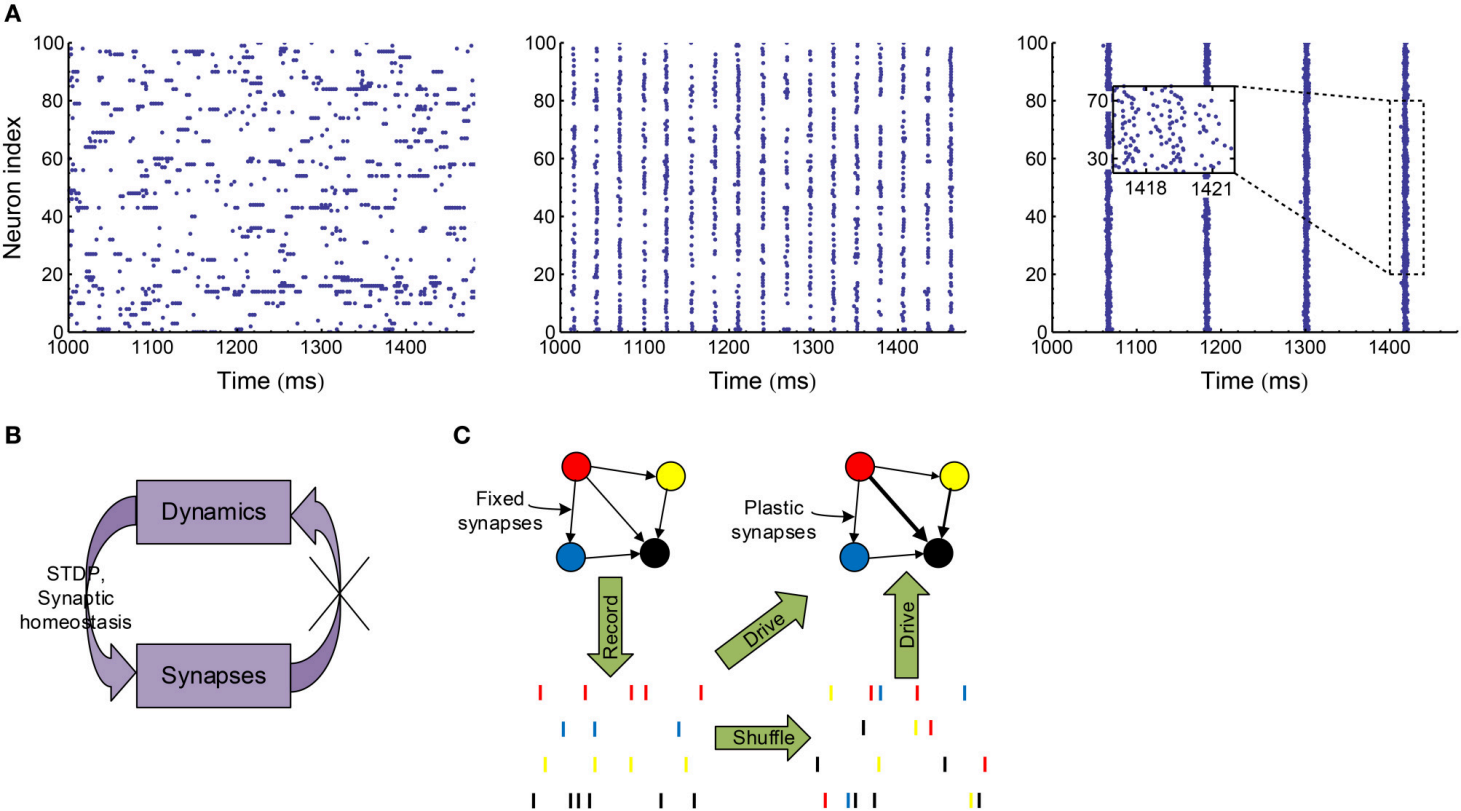
**Overview of our research. (A)** Spike patterns of our LIF network (Section 2.3) at asynchronous (left, $\tau_{d}^{I}=3\text{ ms}$), weak synchronous (middle, $\tau_{d}^{I}=7\text{ ms}$) and synchronously bursting (right, $\tau_{d}^{I}=14\text{ ms}$) states. In weak synchronous states (middle), a neuron usually fires no more than one spike in a synchronous event; and if a neuron fires in a synchronous event, it may be silent in another one. In synchronously bursting states (right), a neuron typically fires more than one spikes burstly in a synchronous event. **(B)** In a plastic network, dynamics and synapses interact and co-evolve with each other. We would like to cut this loop to only investigate the influence of dynamics onto efficacy variability under STDP and synaptic homeostasis without worrying about the change of dynamics caused by synaptic changes. **(C)** To achieve the strategy shown in **(B)**, we first record the spike patterns of the LIF network when the synapses are fixed, then shuffle the spike patterns with a variety of methods to change specific pattern statistics, and at last let the neurons in the network fire according to the recorded or shuffled patterns with STDP and synaptic homeostasis being imposed onto the synapses.

To manifest this idea and also get understanding on the efficacy variability in biologically plausible spike patterns, in this paper, we implement a variety of spike shuffling methods to the spike patterns self-organized by an excitatory-inhibitory LIF network model. It is known that the LIF network can generate synchronized oscillations through two mechanisms (Tiesinga and Sejnowski, 2009; Buzsáki and Wang, 2012), which may depend on the excitatory and inhibitory synaptic time scale τ_*E*_ and τ_*I*_ (Brunel and Wang, 2003). When τ_*E*_ ≪ τ_*I*_, oscillations come from the interaction of the excitatory and inhibitory populations (E-I mechanism): neuronal firing is first driven up by fast excitation, and then dragged down by slow inhibition; when the excitation driving the interneurons wanes, the network recovers from inhibition and the next oscillation cycle starts. When τ_*E*_ ≫ τ_*I*_, oscillations come from the interaction within the inhibitory population (I-I mechanism): in this case, the excitation driving the inhibitory interneurons can be regarded as constant in a relatively small time scale, and the interneurons may synchronously oscillate due to the interactions among them (Wang and Buzsáki, 1996; Brunel and Hakim, 1999), which in turn entrains the excitatory population into oscillation. In this paper, we focus on the synchronized oscillation induced by E-I mechanism, which is suggested to be the mechanism of the fast oscillation in the cortex (Salkoff et al., 2015), the hippocampal ripple oscillations (Stark et al., 2014), and the spike-and-wave electroencephalography (EEG) pattern observed in absence seizures (Destexhe, 2007, 2008). To do this, we fixed the time scale of the excitatory synapses, and increased the decay time scale $\tau_{d}^{I}$ (see Equation 8) of the inhibitory synaptic currents; and then found this network transits from asynchronous state to weak synchronous state and at last to synchronous bursting state (Figure 3A). In reality, the dynamics of a plastic network co-evolve with the synaptic weights. To only investigate the influence of the network dynamics onto the efficacy variability without worrying about the feedback to network dynamics from synaptic changes (Figure 3B), we take the following stategy in this paper (Figure 3C): We first record the spike patterns of the excitatory population of the LIF network, then shuffle the recorded spike patterns using different methods to change different statistical features, and at last evolve the E-E links under STDP (Figure 1B) and synaptic homeostasis (Figure 1C) when the excitatory population are supposed to fire according to the recorded or shuffled spike patterns. By comparing the statistics of the patterns as well as the efficacy variability under the patterns before and after implementing a spike shuffling method, we can gain understanding on how different aspects of the pattern structure may influence the efficacy variability.

We have three aims in this paper. Firstly, we would like to develop a systematic spike-shuffling approach to alter the statistical features of a given spike pattern. Secondly, we will apply this approach to investigate how different pattern statistics (Figure 1A) influence the efficacy variability under STDP and synaptic homeostasis (Figures 1B,C) in the spike patterns self-organized by the LIF network. Thirdly, we will estimate the contributions of different pattern statistics to the efficacy variability under the spike patterns generated by our LIF network, both in asynchronous and synchronous states. For example, we would like to know which spike pattern statistics influences significantly to the efficacy variability, and which statistics is the main reason for the change of the efficacy variability with $\tau_{d}^{I}$.

In the Results section, we will first illustrate the statistics of the spike patterns generated by the LIF network (Section 3.1), and then study the impact of the four statistical features (Figure 1A) onto the efficacy variability by implementing spike shuffling methods onto the spike patterns in both asynchronous and synchronous states (Sections 3.2–3.4). Finally, we will understand the contributions of different pattern statistics to the efficacy variability under these spike patterns (Section 3.5). In the Discussion section, we will discuss the connections of our results to known theoretical and experimental results. We compare the results of this paper with the results of our previous paper (Bi and Zhou, 2016) in Supplementary Materials Section S5, and find their consistency.

## 2. Materials and methods

### 2.1. The definition of efficacy variability

Suppose the weights of a set of synapses W are to be changed by almost the same value during a plasticity process to make the network function normally. We run the plasticity process on the network for several trials, and construct a matrix Δ**W**, each column of which represents the synaptic changes of W in one trial at a given time point *t*, and different columns represent different trials. To quantify the variability of synaptic changes during plasticity, we define *efficacy variability* of W at time *t* to be the variance of the elements of the matrix Δ**W**, i.e., Var_*S, T*_(Δ**W**). Here, the subscript *S* represents integrating over row index, i.e., structural index, and *T* represents integrating over column index, i.e., trial index. Using the law of total variance (Weiss, 2005), it can be shown (see Section 2.1 in our previous paper Bi and Zhou, 2016) that

(2) $$\begin{array}{r} {\text{Var}}_{S,T}\left(\Delta \text{W}\right)={\text{Var}}_{S}\left({\text{E}}_{T}\left(\Delta \text{W}\right)\right)+{\text{E}}_{S}\left({\text{Var}}_{T}\left(\Delta \text{W}\right)\right), \end{array}$$

and that

(3) $$\begin{array}{r} {\text{Var}}_{S,T}\left(\Delta \text{W}\right)={\text{Var}}_{T}\left({\text{E}}_{S}\left(\Delta \text{W}\right)\right)+{\text{E}}_{T}\left({\text{Var}}_{S}\left(\Delta \text{W}\right)\right), \end{array}$$

with E and Var representing mean and variance respectively.

In Equation (2), *E*_*T*_(Δ**W**) represents the trial expectations of the changes of all the synapses in W; and Var_*S*_(*E*_*T*_(Δ**W**)) is the variance of these trial expectations, representing DriftV. Var_*T*_(Δ**W**) represents the trial-to-trial variances caused by diffusion, and *E*_*S*_(Var_*T*_(Δ**W**)) is the average of these variance over all the synapses, representing DiffV. This equation is the formal writing of Equation (1) in the introduction.

In Equation (3), Var_*T*_(*E*_*S*_(Δ**W**)) represents the trial-to-trial variability of the mean synaptic change of the whole network. But a real biological process only allows a single trial, so this trial-to-trial variability cannot contribute to biological functions except for individual differences. Additionally, in this paper, we aim to understand the influence of spike pattern structures onto efficacy variability, so the firing rates and second-order statistics of spike patterns need to be controlled in our model. In this theoretical context, under STDP, Var_*T*_(*E*_*S*_(Δ**W**)) is presumably of the order of O(1∕|W|), with |W| being the number of synapses in W. So when |W| is large enough, Equation (3) becomes

(4) $$\begin{array}{r} {\text{Var}}_{S,T}\left(\Delta \text{W}\right)\approx {\text{E}}_{T}\left({\text{Var}}_{S}\left(\Delta \text{W}\right)\right). \end{array}$$

This tells that we can approximate the efficacy variability by the trial average of the variance of the synaptic changes in the network, which, as shown in Figure 2, may have strong biological implications. In this paper, we use *E*_*T*_(Var_*S*_(Δ**W**)) to quantify the efficacy variability in our simulations. As we show in Supplementary Figure 1, Var_*T*_(*E*_*S*_(Δ**W**)) is negligible comparing to *E*_*T*_(Var_*S*_(Δ**W**)), so *E*_*T*_(Var_*S*_(Δ**W**)) is indeed nearly the same with the full version Var_*S, T*_(Δ**W**) in our simulations.

### 2.2. STDP and synaptic homeostasis

The STDP updating caused by a pair of pre- and post-synaptic spike at *t*_*pre*_ and *t*_*post*_ is

(5) $$\begin{array}{r} \Delta w\left(t_{pre},t_{post}\right)=\left\{ \begin{array}{cc} A_{p}\text{exp}\left(-\frac{t_{post}-\left(t_{pre}+\tau_{delay}\right)}{\tau_{STDP}}\right),\; & t_{post}>t_{pre}+\tau_{delay}\\ -A_{d}\text{exp}\left(-\frac{\left(t_{pre}+\tau_{delay}\right)-t_{post}}{\tau_{STDP}}\right),\; & t_{post}<t_{pre}+\tau_{delay} \end{array}\right. \end{array}$$

with τ_*delay*_ being the axonal delay. The contributions of all pairs of pre- and post-synaptic spikes are added together. τ_*STDP*_ = 20 ms, τ_*delay*_ = 1 ms throughout the paper, and *A*_*p*_ = *A*_*d*_ = 1 by default.

As explained in Figures 3B,C, we did not directly embed STDP and synaptic homeostasis into the self-organizing dynamics of the LIF network, but instead evolved the E-E links under STDP and synaptic homeostasis according to the recorded or shuffled spike patterns of the LIF network with fixed synaptic weights. We recorded the variance of synaptic weights during this evolution every 1 s of biological time, and before each recording, we implemented synaptic homeostasis onto the synapses within excitatory population as

(6) $$\begin{array}{r} w_{ab}\to w_{ab}+\left(w_{bound}-\frac{1}{N_{a}}\overset{N_{a}}{\underset{c=1}{\sum }}w_{ac}\right), \end{array}$$

with *w*_*ab*_ being the weight of the synapse from excitatory neuron *b* to *a*, *N*_*a*_ being the excitatory in-degree of the *a*th neuron, and *w*_*bound*_ = 0 being the ground line of synaptic homeostasis. In this way, the mean excitatory synaptic input to each excitatory neuron was fixed at *w*_*bound*_ = 0 before each recording.

### 2.3. The LIF neuronal network

The network consists of 2000 excitatory and 500 inhibitory conductance-based LIF neurons, with the links being randomly connected with probability 0.2. Each neuron in the network also receives excitatory external inputs.

The dynamics of the sub-threshold membrane voltage $V_{i}^{\alpha }$ of the *i*th neuron in the αth (α = *E, I* representing excitatory or inhibitory) population is (see e.g., Brunel and Wang, 2003)

(7) $$\begin{array}{l} C^{\alpha }\frac{dV_{i}^{\alpha }(t)}{dt}=g_{L}^{\alpha }(V_{leak}-V_{i}^{\alpha }(t))+[g^{\alpha ,ext}s_{i}^{\alpha ,ext}(t)\\ \;+\;g^{\alpha E}\sum_{j}A_{ij}^{\alpha E}s_{ij}^{\alpha E}(t)](E_{E}-V_{i}^{\alpha }(t))\\ \;+\;g^{\alpha I}\sum_{j}A_{ij}^{\alpha I}s_{ij}^{\alpha I}(t)(E_{I}-V_{i}^{\alpha }(t)), \end{array}$$

with *C*^α^ being the membrane capacity, $g_{L}^{\alpha }$ the leakage conductance, *V*_*leak*_ the leakage voltage, *g*^α, *ext*^ the strength of the synapses from external inputs to the neurons in the αth neuronal population, $s_{i}^{\alpha ,ext}$ the synaptic conductance of unit synaptic strength from the external inputs, *g*^αβ^ (β = *E, I*) the strength of the synapses from the βth population to the αth population, $s_{ij}^{\alpha \beta }$ the synaptic conductance of unit synaptic strength from the *j*th neuron in the βth population connected to the *i*th neuron in the αth population, $A_{ij}^{\alpha \beta }=1,0$ indicating whether the *j*th neuron in the βth population connects to the *i*th neuron in the αth population or not, and *E*_*E*_ (*E*_*I*_) being the inverse voltage for the excitatory (inhibitory) synaptic current. The membrane voltage $V_{i}^{\alpha }$ is reset to *V*_*r*_ as soon as it crosses threshold θ, and will stay at *V*_*r*_ for a refractory period $\tau_{ref}^{\alpha }$ after this reset.

The dynamics of the synaptic conductance of unit synaptic strength $s_{ij}^{\alpha \beta }$ follows (Brunel and Wang, 2003)

(8) $$\begin{array}{l} s_{ij}^{\alpha \beta }(t)=\sum_{k}\Theta (t-t_{k}-\tau_{delay})\frac{\tau_{m}^{\alpha }}{\tau_{d}^{\alpha }-\tau_{r}^{\alpha }}[\exp(-\frac{t-t_{k}-\tau_{delay}}{\tau_{d}^{\alpha }})\\ \;-\exp(-\frac{t-t_{k}-\tau_{delay}}{\tau_{r}^{\alpha }})], \end{array}$$

with {*t*_1_, *t*_2_, ⋯ } being the spike train that the synapse receives, $\tau_{m}^{\alpha }\equiv C^{\alpha }∕g_{L}^{\alpha }$ the membrane time constant of the αth population, $\tau_{r}^{\alpha }$ ($\tau_{d}^{\alpha }$) the rising (decaying) time scale of the synaptic conductance in response to an incoming spike, and τ_*delay*_ = 1 ms the axonal delay. The normalization factor $\frac{\tau_{m}^{\alpha }}{\tau_{d}^{\alpha }-\tau_{r}^{\alpha }}$ is chosen so that varying the synaptic time constant does not affect the time integral of a postsynaptic current (Brunel and Wang, 2003). The dynamics of $s_{i}^{\alpha ,ext}\left(t\right)$ is determined by the external input spike trains, with the other parameters being that same as those for $s_{ij}^{\alpha E}\left(t\right)$.

In our simulations, $g_{L}^{E}=g_{L}^{I}=10\text{ nS}$, $C^{E}=20\text{ ms}\cdot g_{L}^{E}$, $C^{I}=10\text{ ms}\cdot g_{L}^{I}$; *E*^*E*^ = 0 mV, $V_{leak}=E^{I}=-70\text{ mV}$; *g*^*EE*^ = 0.4 nS, *g*^*EI*^ = 5.8 nS, *g*^*IE*^ = 0.74 nS, *g*^*II*^ = 9.6 nS; *V*_*r*_ = −60 mV, θ = −50 mV; $\tau_{ref}^{E}=2\text{ ms}$, $\tau_{ref}^{I}=1\text{ ms}$; $\tau_{r}^{E}=\tau_{r}^{I}=0.5\text{ ms}$, $\tau_{d}^{E}=4\text{ ms}$. The decaying time constant $\tau_{d}^{I}$ of all the inhibitory synapses are the same in a simulation trial, but may differ in different trials, resulting in different network dynamics (see Figure 3A). In a single trial, $\tau_{d}^{I}$ may take one value in the 12 integers from 3 to 14 ms. Each neuron also receives 1000 Hz external Poisson input, with external conductance *g*^*E, ext*^ = *c* × 0.53 nS, *g*^*I, ext*^ = *c* × 0.75nS, with *c* being a coefficient whose value depends on $\tau_{d}^{I}$. To keep the excitatory population almost at 20 Hz for different trials, we choose *c* to be 3.1459, 3.28212, 3.38699, 3.46922, 1.60585, 1.46867, 1.27884, 1.04738, 0.82432, 0.616324, 0.410436, 0.323722 for $\tau_{d}^{I}$ as integer values from 3 to 14 ms (see Supplementary Materials Section S2 for more details on how we chose *c*). Simulations were performed using a second order Runge–Kutta scheme with fixed time step δ*t* = 0.05 ms; and an interpolation scheme was also used for the determination of the firing times of the neurons (Hansel et al., 1998).

The purpose of this work is to understand how the dynamic patterns influence the efficacy variability, instead of how the dynamic properties change with model parameters; so averaging configurations of the random LIF networks does not help to gain more insight to the problem being addressed, only increasing complexity. Therefore, our study focused on a single typical network configuration, except that we chose different initial states and seeds of random generators for different trials, which resulted in trial-to-trial variability. We did check our results using other network configurations, and found qualitatively the same results.

### 2.4. Fitting *c*_*diffv*_ and *c*_*driftv*_

Because of the additive nature of our STDP model, DiffV ∝ *t* and DriftV ∝ *t*^2^. Therefore, the time evolution of the efficacy variance *v*(*t*) can be written as $v\left(t\right)=c_{diffv}t+c_{driftv}t^{2}$ in a long run, with *c*_*diffv*_ and *c*_*driftv*_ respectively quantifying the strength of DiffV and DriftV. To estimate the values of *c*_*diffv*_ and *c*_*driftv*_, we let the excitatory population fire according to the recorded or shuffled spike pattern, with STDP and synaptic homeostasis starting after the initial 2 s of transient period. We then recorded the efficacy variance at time 15, 16,…, 39 s after the transient period, and did linear regression using the formula *v*(*t*)∕*t* = *c*_*diffv*_+*c*_*driftv*_*t*. The estimated values and standard errors of *c*_*diffv*_ and *c*_*driftv*_ were then used to plot the relevant panels in **Figures 8**, **9**. See Supplementary Materials Section S3 for more details on the fitting procedure and the goodness of fit.

### 2.5. Spike pattern analysis

Here are the methods we used to analyze the statistics of the spike patterns of the LIF network, both originally recorded and after being shuffled. Each trial of our simulation lasted for 41 s biological time, with the first 2 s regarded as transient period and excluded from the following analysis.

#### 2.5.1. Analyzing the statistics of synchronous firing

For spike patterns in synchronous states ($\tau_{d}^{I}\geq 7\text{ ms}$), *synchronous events* are numerically defined as follows: the firing rate of the excitatory population is first calculated in bins of 0.1 ms, then filtered using Gaussian window of σ_*window*_ = 1 ms, and *synchronous events* are then defined as the sequential bins in which the filtered rates are above a threshold 1 Hz. The size of the Gaussian window and the value of the threshold are chosen so that the duration of a synchronous event is large enough to include nearly all the spikes that spurt synchronously (see Figure 3A, middle and right panels), and at the same time as small as possible. It is possible that some spikes are not included in any synchronous event, and these spikes will be excluded from the statistical analysis of synchronous events.

The firing profiles of the neurons in synchronous events shown in **Figure 10E** are calculated as follows. For a synchronous spike pattern, we first order the firing rates of all the neurons (2000 in total) from low to high, and then put the 290–299th neurons into “low rate” bracket, the 1190–1199th neurons into “middle rate” bracket, 1990–1999th into “high rate” bracket, and all the neurons into “whole” bracket. Suppose *r*_*m,i,s*_(*t*) to be the firing rate (calculated by counting spike numbers within bins of 0.1 ms) of the *i*th neuron in the *m*th bracket in the *s*th synchronous event, then the firing profile of the *m*th bracket is defined as

(9) $$\begin{array}{rcl} {\bar{r}}_{m}\left(t\right)=\frac{1}{N_{i}N_{s}}\underset{i,s}{\sum }r_{m,i,s}\left(t+t_{s}\right)\;\\ m & = & \text{low rate},\text{middle rate},\text{high rate},\text{whole}, \end{array}$$

with *N*_*i*_ being the number of neurons in the bracket, *N*_*s*_ being the number of synchronous events, and *t*_*s*_ being the middle time (i.e., the mean time of all the spikes) of the *s*th synchronous event. The idea of this equation is to first translationally move all the synchronous events so that the middle times of these synchronous events are all located at 0, and then average the firing rates in these synchronous events for all the neurons in the same bracket.

In **Figure 7A**, we use *p* and τ_*cross*_ to quantify the mean strength and duration of synchronous events. *p* is defined as the mean spike number per neuron per synchronous event. To calculate τ_*cross*_, we use the firing profile ${\bar{r}}_{whole}\left(t\right)$ defined by Equation (9), and τ_*cross*_ is then defined as the duration between the two times at which ${\bar{r}}_{whole}\left(t\right)$ drops below 10% of its peak value from its peak time for the first time, along both the positive and negative directions.

#### 2.5.2. Analyzing the statistics of auto-correlation structure

For spike patterns in asynchronous states (τ_*d, I*_ ≤ 6 ms), auto-correlation structure is quantified by the mean coefficient of variance (*CV*, which is the ratio of the standard deviation and mean of the inter-spike intervals) over all the spike trains, representing the burstiness/regularity of these spike trains.

**Figure 4:**
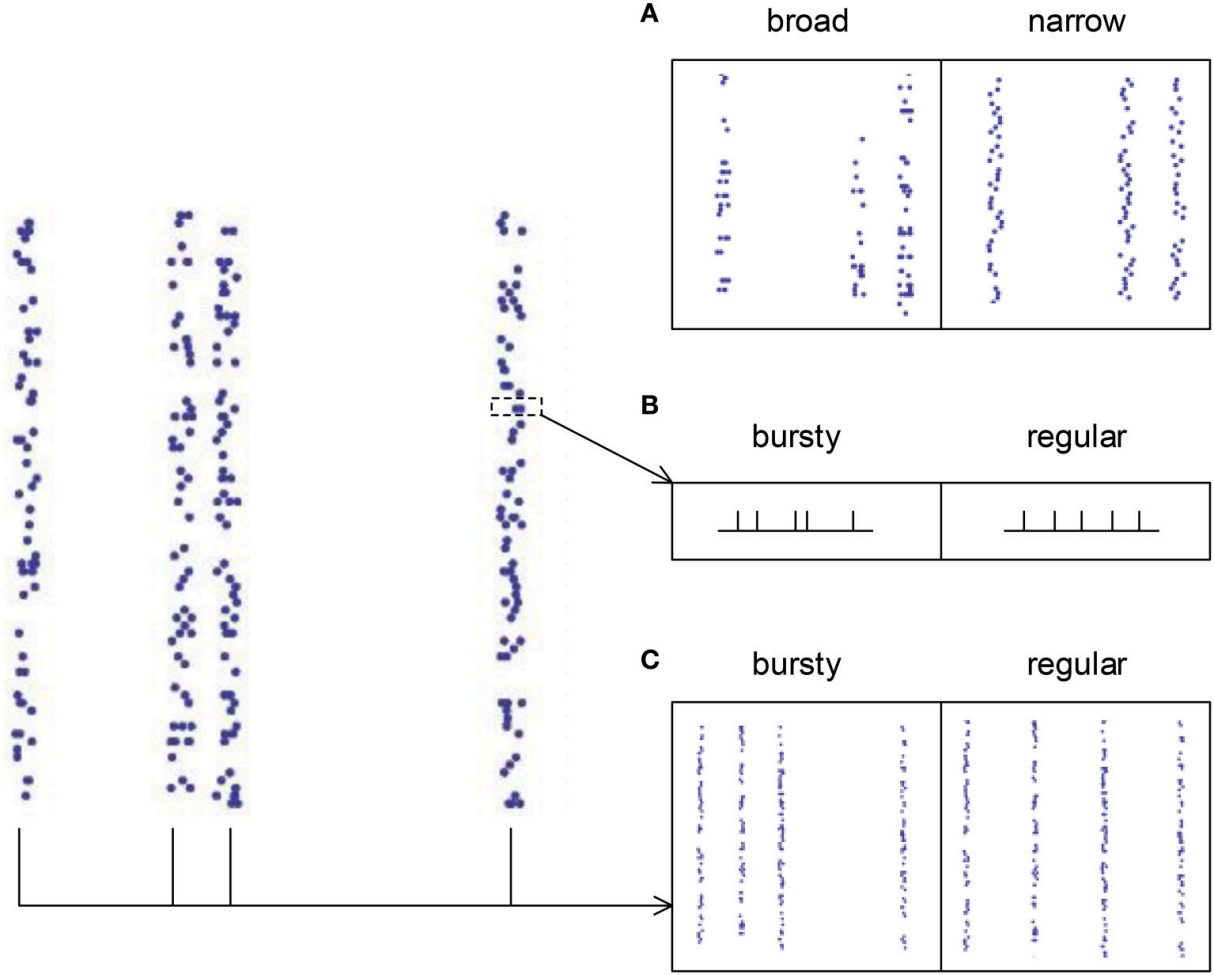
**The three types of auto-correlation structure we consider under synchronous firing. (A)** The broadness of the distribution of the spike numbers a neuron fires in different synchronous events. Note that in the left panel, a neuron fires quite different number of spikes during different synchronous events; while in the right panel, the spike numbers of a neuron during different synchronous events are almost the same. **(B)** The burstiness/regularity of the pieces of spike trains within synchronous events. **(C)** The burstiness/regularity of the occurrence of synchronous events.

For spike patterns in synchronous states (τ_*d, I*_ ≥ 7 ms), auto-correlation structure is separated into three aspects (Figure 4): (1) the broadness of the distribution of the spike numbers a neuron fires in different synchronous events (AT_SpikeNum_), (2) the burstiness/regularity of pieces of spike trains within synchronous events (AT_WithinEvent_), and (3) the burstiness/regularity of the occurrence of synchronous events (AT_events_). AT_SpikeNum_ is quantified by the variance of the distribution of the spike numbers a neuron fires in different synchronous events. AT_WithinEvent_ is quantified by *CV*_*WithinEvent*_, which is calculated by averaging over the *CV* values of the pieces of spike trains that contain more than 2 spikes in synchronous events. AT_events_ is quantified by the *CV* of the middle times (i.e., the mean time of all the spikes) of the synchronous events.

#### 2.5.3. Analyzing the statistics of heterogeneity of rates

Heterogeneity of rates means the heterogeneity of time-averaged firing rates for different neurons in the spike patterns. It is quantified by the variance of the time-averaged firing rates.

#### 2.5.4. Analyzing the statistics of heterogeneity of cross-correlations

We define the *unit cross-correlation* between neuron *a* and neuron *b* to be *C*_*ab*_(τ) = 〈*r*_*a*_(*t*)*r*_*b*_(*t* + τ)〉∕(〈*r*_*a*_(*t*)〉〈*r*_*b*_(*t*)〉), with *r*_*a*_(*t*) being the firing rate of the *a*th neuron, and 〈·〉 representing averaging over time. In this paper, heterogeneity of cross-correlations typically means that the a post-synaptic neuron has different unit cross-correlations with its different pre-synaptic neurons. It reflects that different pre-synaptic neurons tend to fire at different times relative to a post-synaptic spike.

To quantify the strength of heterogeneity of cross-correlations, we define *Index of heterogeneity of cross-correlations (HCC)* to quantify ${\text{E}}_{b}\left({\text{Var}}_{a\in \partial b}\left(\int_{-\infty }^{\infty }H\left(\tau \right)C_{ab}\left(\tau \right)\text{d}\tau \right)\right)$, with ∂*b* representing all the pre-synaptic neurons of *b*, and *H*(τ) being the STDP time window (see Equation 5). It is estimated as follows: for a synapse from neuron *a* to neuron *b*, we denote Δ*n*_*a*→*b*_ as the synaptic change per unit time under STDP alone (without considering synaptic homeostasis), and denote $\Delta m_{a\to b}=\frac{\Delta n_{a\to b}}{r_{a}r_{b}}$ (with *r*_*a*_ and *r*_*b*_ being the time-averaged firing rates). The index of HCC is then calculated as *E*_*b*_(Var_*a*∈∂*b*_(Δ*m*_*a*→*b*_)).

### 2.6. Spike shuffling methods

**Figure 5:**
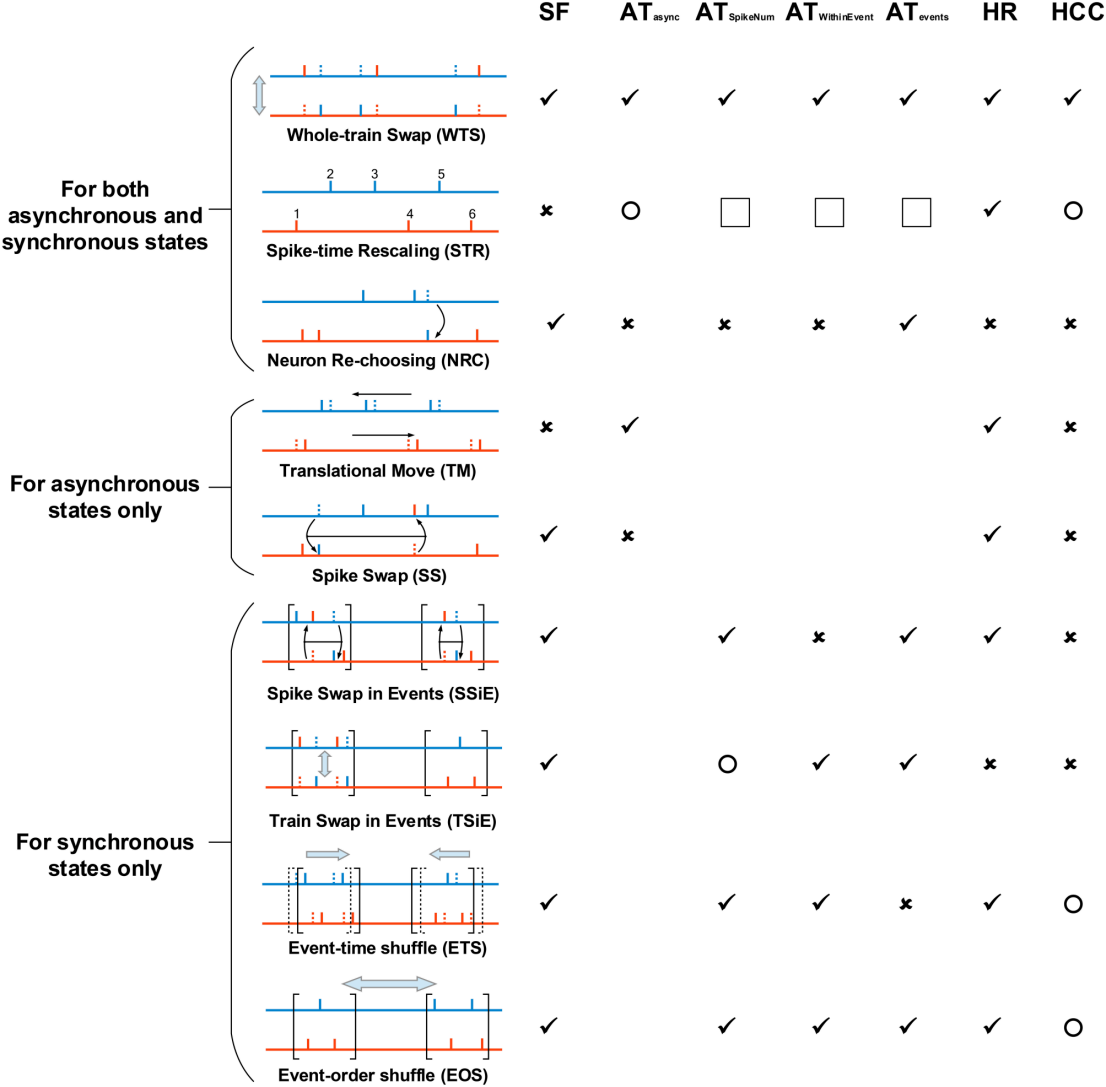
**The spike shuffling methods we use to study the spike patterns of our LIF network, and their influences onto spike pattern structures**. The most left panel explains the spike shuffling methods we use to treat spike patterns, and the rest columns in the right show the influence of these spike shuffling methods onto synchronous firing (SF), auto-correlation structure (AT), heterogeneity of rates (HR) and heterogeneity of cross-correlations (HCC). We consider three types of auto-correlation structure for synchronous states AT_SpikeNum_, AT_WithinEvent_ and AT_events_ (see Figure 4), and the auto-correlation structure under asynchronous states are shortly represented by AT_async_. ✓ means that a shuffling method keeps a pattern structure unchanged; × means that a shuffling method *destroys* a pattern structure. Here, “destroy” has a sense of “completely randomize.” For SF, “destroy” means that there is no time fluctuation of population firing rate. For AT_async_, it means that the spike train of the *a*th neuron can be regarded as an inhomogeneous Poisson process with rate *r*_*a*_(*t*) = *r*_*a*_*x*(*t*), with *r*_*a*_ being the time-averaged firing rate, and *x*(*t*) being the same for all the neurons. Because of the weak fluctuation of the population firing rate in asynchronous states, this also makes *CV* ≈ 1. For AT_SpikeNum_, it means that the spike numbers of the neurons within a synchronous event follows Poisson distribution of parameter *p*, with *p* being the mean spike number per neuron within the synchronous event. For AT_WithinEvent_, it means that the spike train of the *a*th neuron can be regarded as an inhomogeneous Poisson process with rate *r*_*a*_(*t*) = *r*_*a*_*x*(*t*), with *r*_*a*_ being the time-averaged firing rate, and *x*(*t*) being the same for all the neurons. For AT_events_, it means that the occurrence of synchronous events can be regarded as a Poisson process. For HR, it means that the time-averaged firing rates of all the neurons are the same. For HCC, it means that the unit cross-correlations $C_{ab}\left(\tau \right)=\frac{\langle r_{a}\left(t\right)r_{b}\left(t+\tau \right)\rangle }{\langle r_{a}\left(t\right)\rangle \langle r_{b}\left(t\right)\rangle }$ (with *r*_*a*_(*t*) representing the firing rate of the *a*th neuron) are the same for different neuronal pairs. ◯ means that a shuffling method may change, but does not “completely randomizes,” a pattern structure. Note that STR completely flattens the rate fluctuation with time, so AT_SpikeNum_, AT_WithinEvent_ and AT_events_ are not applicable to the synchronous spike patterns after STR (indicated by the squares in the figure). However, note that AT_SpikeNum_ and AT_WithinEvent_ of the spike patterns before STR can influence the burstiness/regularity of the asynchronous spike patterns after STR. All these spike shuffling methods are further illustrated in Supplementary Movie.

Here we list out the spike shuffling methods we used for spike patterns in asynchronous states, and discuss how each of them changes spike pattern statistics. We use different spike shuffling methods for the spike patterns in asynchronous and synchronous states, because of the sharp difference of their pattern structure. Here we first list out the shuffling methods that are used for both asynchronous and synchronous states, then list out those only used for asynchronous states, and finally those only for synchronous states. See Figure 5 and Supplementary Movie for illustration of these spike shuffling methods.

#### 2.6.1. Spike shuffling methods for both asynchronous and synchronous states

##### 2.6.1.1. Whole-train swap (WTS)

This method randomly shuffles the neuronal indexes of spike trains in the pattern. For example, if we denote T_*a*_ to be the spike train of the *a*th neuron, then the whole spike pattern can be denoted as a set of neuron-train pairs {(*a*, T_*a*_), (*b*, T_*b*_), (*c*, T_*c*_), ⋯ }; then after WTS, the spike pattern may become {(*a*, T_*c*_), (*b*, T_*a*_), (*c*, T_*b*_), ⋯ }. By definition, WTS keeps all the statistics of a spike pattern, but destroys the possible correlation between the spike trains and the structure of the underlying neuronal network. To get rid of this pattern-network coupling thereby focusing on the influence to the efficacy variability by spike pattern statistics (see the discussions in Section 3.2), we treated all the recorded spike patterns by WTS before any other shuffling method.

##### 2.6.1.2. Spike-time rescaling (STR)

The idea of this method is that the spike times are first projected to the rescaled time defined as the accumulative function of the population firing rate *r*(*t*)

(10) $$\begin{array}{r} \Lambda \left(t\right)=\int_{0}^{t}r\left(s\right)\text{d}s, \end{array}$$

and then are projected back to the normal time using $\Lambda_{0}^{-1}\left(s\right)$, where Λ_0_(*t*) is the linear function connecting (0, 0) with (*T*, Λ(*T*)), with *T* being the duration of the spike pattern. In this way, inter-spike intervals are rescaled according to the population firing rate, so that the population firing rate is kept constant in the spike pattern after shuffling. Technically, STR is realized by first ordering all the *M* spikes in the pattern, then setting the time of the *i*th spike at *iT*∕*M*. By definition, this shuffling method flattens population firing rate, while conserving the time-averaged firing rate of each neuron. As it keeps the order of spikes, the burstiness/regularity of spike trains and cross-correlations between spike trains in the original pattern can be, to some extent, kept in the pattern after shuffling, especially if the rate fluctuation in the original spike pattern is weak.

##### 2.6.1.3. Neuron re-choosing (NRC)

In this method, each spike in the pattern is assigned to a randomly selected neuron. When the population size is large, this method makes all the neurons to fire as Poisson processes with equal time-dependent firing rate (i.e., *r*_*a*_(*t*) = *r*_*b*_(*t*) for two different neurons *a* and *b*). NRC destroys auto-correlation structure, heterogeneity of cross-correlations and heterogeneity of firing rates, but keeps the time fluctuation of population firing rate.

Note that under synchronous states, NRC also changes the distribution of the spike numbers a neuron fires in different synchronous events (i.e., AT_SpikeNum_). When the population size is large, this distribution after NRC is a Poisson distribution with parameter *p*, with *p* being mean spike number per neuron within a synchronous event (i.e., the strength of the synchronous event).

#### 2.6.2. Spike shuffling methods only for asynchronous states

##### 2.6.2.1. Translational move (TM)

In this method, each spike train is translationally moved by a random displacement, and periodic boundary condition is used to deal with the spikes which are moved out of the boundaries of time. By definition, TM keeps the auto-correlation structure of spike trains, and the time-averaged firing rate of each neuron. It flattens the cross-correlations between any pair of spike trains, thereby destroying both synchronous firing and heterogeneity of cross-correlations.

##### 2.6.2.2. Spike swap (SS)

The idea of this method is to swap pairs of randomly chosen spikes of different neurons many times. A spike pattern can be denoted as a set of number pairs {(*a, t*_1_), (*b, t*_2_), (*c, t*_3_), ⋯ }, with *a, b, c*, ⋯ being neuronal indexes, and *t*_1_, *t*_2_, *t*_3_, ⋯ being spike times. Technically, SS shuffles the order of the first fields of these number pairs, so that the spike pattern after SS may be {(*b, t*_1_), (*c, t*_2_), (*a, t*_3_), ⋯ }. SS does not change the spike number of a neuron, but randomizes the occurrence of these spikes. When the size of the network is large, the probability that a neuron fires near time *t* is proportional to the global firing rate at *t*, and the times of different spikes are independent with each other. Because of this, SS makes the spike trains Poisson processes, and the rate fluctuations of all these spike trains are simultaneously time-modulated: i.e., the firing rate of the *a*th spike train can be written as *r*_*a*_(*t*) = *r*_*a*_*x*(*t*), with *r*_*a*_ being the time-averaged firing rate, and *x*(*t*) being the same for all the spike trains. By definition, SS destroys auto-correlation structure and heterogeneity of cross-correlations, but keeps the time fluctuation of population firing rate and the time-averaged firing rate of each neuron.

#### 2.6.3. Spike shuffling methods only for synchronous states

##### 2.6.3.1. Spike swap in events (SSiE)

The idea of SSiE is to swap pairs of randomly chosen spikes of different neurons many times, with each pair of chosen spikes being within the same synchronous event. It can be understood as implementing SS (see Section 2.6.2.2) onto each synchronous event in the spike pattern. Our numeric definition of “synchronous event” is presented in Section 2.5. By definition, SSiE keeps the spike number of a neuron within a synchronous event. And the same as SS, SSiE also makes the neurons to fire like Poisson processes when the size of the neuronal population is large, and the rate fluctuation of all the spike trains are simultaneously time-modulated. By definition, SSiE destroys heterogeneity of cross-correlations and AT_WithinEvent_.

##### 2.6.3.2. Train swap in events (TSiE)

The idea of TSiE is to swap the pieces of spike trains of randomly selected neurons pairs in the same synchronous event many times, and the pieces of spike pattern in different synchronous events are shuffled independently. By definition, TSiE destroys heterogeneity of rates and heterogeneity of cross-correlations, but keeps AT_WithinEvent_. The spike number distribution per neuron per synchronous event for the whole neuronal population is kept unchanged under TSiE, but the distribution for a single neuron in different synchronous events is changed dramatically.

##### 2.6.3.3. Event-time shuffle (ETS)

The idea of ETS is that all the spikes within the same synchronous events are translationally moved by a random displacement, at the same time (1) avoiding the overlapping of different synchronous events, and (2) keeping the order of synchronous events unchanged. Here, by “avoiding synchronous-events overlapping”, we have two meanings. Firstly, we mean that if a synchronous event S happens in the time interval [*t*_1_, *t*_2_], then no other synchronous events should happen in this interval. Secondly, because of the axonal delay τ_*delay*_, a post-synaptic neuron receives its pre-synaptic spikes in S during the interval [*t*_1_ + τ_*delay*_, *t*_2_ + τ_*delay*_], and we would like the post-synaptic neuron to receive no spikes from another synchronous event between the time that it fires in S and the time that it finishes receiving all its pre-synaptic spikes in S. In a word, these two conditions mean that if a synchronous event happens in the time interval [*t*_1_, *t*_2_], then no other synchronous event should appear within [*t*_1_−τ_*delay*_, *t*_2_], with τ_*delay*_ being the axonal delay. Technically, this is realized by first randomly selecting *N*_*event*_ points in the duration $\left[0,T-\overset{N_{events}}{\underset{i=1}{\sum }}\left(T_{i}+\tau_{delay}\right)\right]$ (with *N*_*event*_ being the number of the synchronous events, *T* being the time duration of the spike pattern, and *T*_*i*_ being the duration of the *i*th synchronous event, see Section 2.5 for the numeric definition of “synchronous event”), then set the beginning time of the *j*th synchronous event at $x_{j}+\overset{j-1}{\underset{i=1}{\sum }}\left(T_{i}+\tau_{delay}\right)$ (with *x*_*j*_ being the *j*th selected points). The reason why we avoid the synchronous-events overlapping is that firstly getting rid of the first constraint above may change AT_WithinEvent_ in some synchronous events, thereby changing the efficacy variability through this side effect; and secondly, our previous paper (Bi and Zhou, 2016) shows that under the second constraint above the change of DriftV caused by *CV*_*events*_ is simple to understand, and we would like to focus on this simple situation here. The reason why we keep the order of synchronous events is that there may be dependencies (on, say, spike times, or spike numbers) between the pieces of spike trains in adjacent synchronous events, and we would like to keep these possible dependencies to the most extent during ETS. Note that ETS not only change AT_event_, but may also change heterogeneity of cross-correlations by changing the STDP interactions of spikes in adjacent synchronous events.

##### 2.6.3.4. Event-order shuffle (EOS)

EOS shuffles the occurrence order of the synchronous events, so that the synchronous events appear at the same time points as in the original pattern, but in a shuffled order. For example, if the mean spike time of the *i*th synchronous event is at ${\bar{t}}_{i}$, then all the synchronous events in the whole spike pattern may be denoted as a set of number pairs $\left\{\left(i,{\bar{t}}_{i}\right),\left(j,{\bar{t}}_{j}\right),\left(k,{\bar{t}}_{k}\right),\cdots \;\right\}$; after EOS, this pattern may become $\left\{\left(i,{\bar{t}}_{k}\right),\left(j,{\bar{t}}_{i}\right),\left(k,{\bar{t}}_{j}\right),\cdots \;\right\}$. EOS destroys possible dependencies (on, say, spike times or spike numbers) between the pieces of spike trains in adjacent synchronous events. By comparing the efficacy variability under spike patterns before and after EOS, we found that such dependencies have little influences onto efficacy variability in our model (Supplementary Figure 9).

## 3. Results

### 3.1. The dynamic patterns of the LIF network

**Figure 6:**
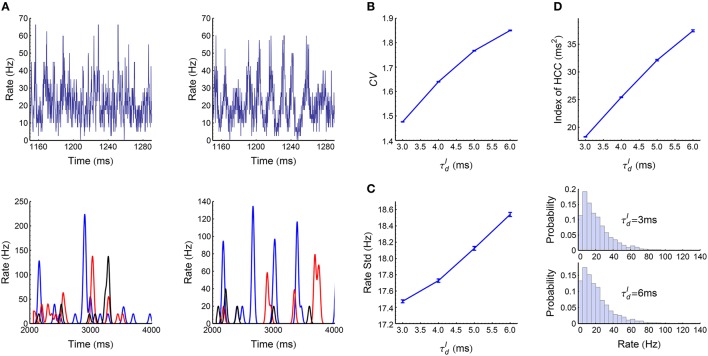
**Statistics of the spike patterns of the LIF network in asynchronous states. (A)** Upper panels: population firing rate as a function of time when $\tau_{d}^{I}=3$ ms (left) and 6 ms (right). Lower panels: firing rates for three example neurons, when $\tau_{d}^{I}=3$ ms (left) and 6 ms (right). We estimated the instantaneous population firing rates using bins of 0.1 ms, and estimated the instantaneous firing rates of individual neurons by convolving their spike trains with a 40-ms-wide Gaussian filter. **(B)** Mean coefficient of variance (*CV*) of all the spike trains in a spike pattern as a function of $\tau_{d}^{I}$. **(C)** Left panel: the standard deviation of population rate as a function of $\tau_{d}^{I}$. Right panel: the distributions of firing rates when $\tau_{d}^{I}=3$ ms and 6 ms. **(D)** The index of heterogeneity of cross-correlations (HCC) (see Section 2.5) as a function of $\tau_{d}^{I}$. **(A)** and the right panel of **(C)** are plotted from one simulation trial, while the other panels are plotted from averaging over 32 simulation trials. Simulation details are explained in Section 2.3. Error bars represent s.e.m.

When the decaying time scale $\tau_{d}^{I}$ of the inhibitory synaptic inputs (Equation 8) is within the range $3\text{ ms}\leq \tau_{d}^{I}\leq 6\text{ ms}$, the LIF network (Section 2.3) works in asynchronous states (Figure 3A). In this case, we found the population firing rate exhibits strong fluctuation with time (Figure 6A, upper panels). The firing rate of individual neurons also fluctuate strongly (Figure 6A, lower panels); and the coefficient of variance (*CV*) is larger than 1 (Figure 6B), reflecting the burstiness of the spike patterns. These features suggest that the network works in chaotic asynchronous states, which may be caused by the unstability of the network dynamics to heterogeneous perturbations (Ostojic, 2014). The firing rates are heavily skewed (Figure 6C), which may be caused by the heterogeneous input degrees and nonlinear current-rate relationship of neurons (Roxin et al., 2011). There also exists non-trivial cross-correlations between neurons in these asynchronous patterns (Figure 6D), which may be caused by connectivity details such as unidirectional connections and input sharing (Ostojic et al., 2009; Trousdale et al., 2012).

**Figure 7:**
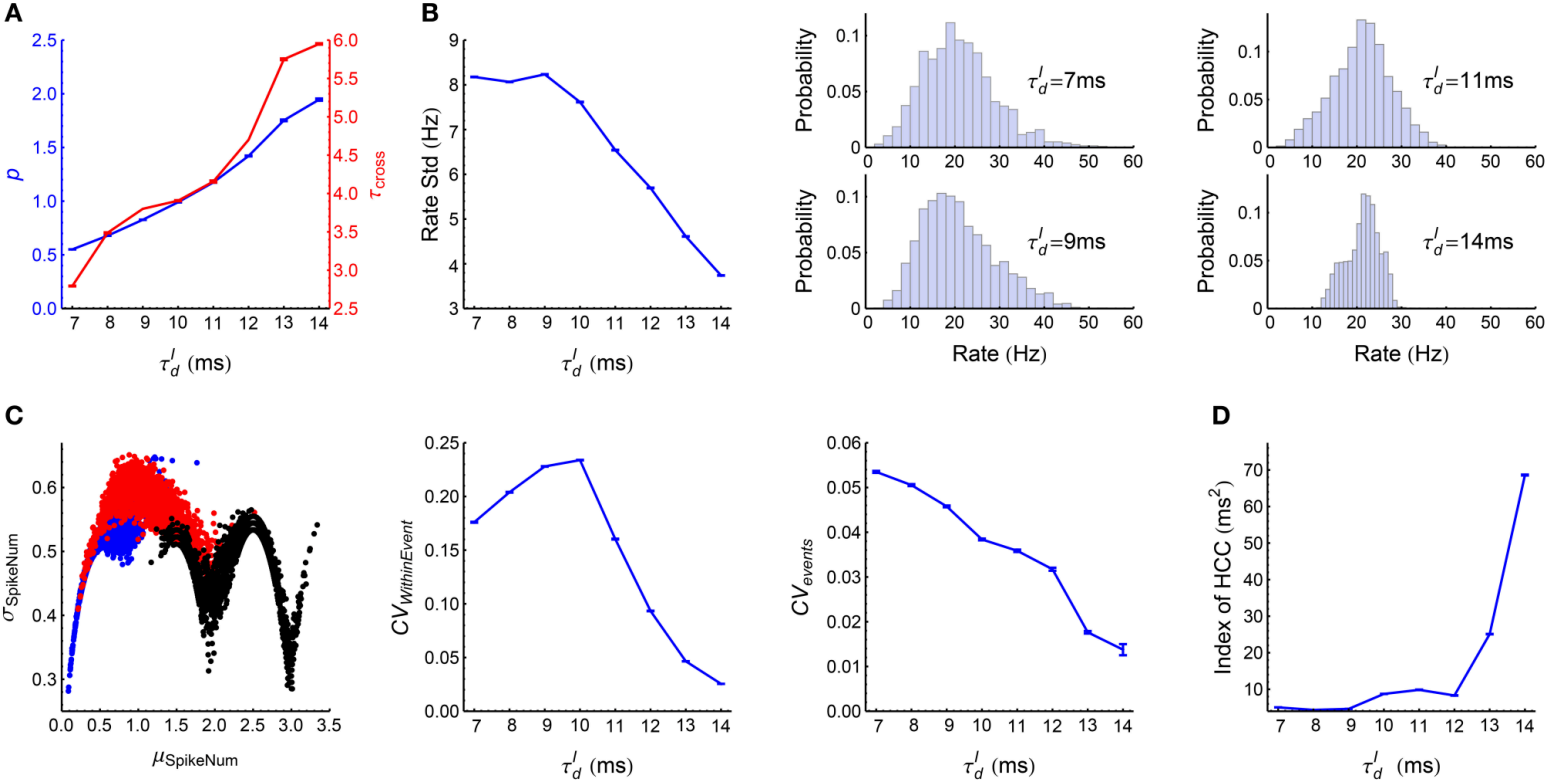
**Statistics of the spike patterns of the LIF network in synchronous states. (A)** The strength *p* and duration τ_*cross*_ of synchronous events as functions of $\tau_{d}^{I}$ (see Section 2.5 for details). **(B)** Left panel: Standard deviation of firing rates as a function of $\tau_{d}^{I}$. Middle and right panels: the distributions of firing rates when $\tau_{d}^{I}$ takes the indicated four values. **(C)** Left panel: σ_*SpikeNum*_ as a function of μ_*SpikeNum*_ when $\tau_{d}^{I}=7$ ms (blue), 10 ms (red) and 14 ms (black), with μ_*SpikeNum*_ and σ_*SpikeNum*_ being the mean and standard deviation of the spike number distribution for a neuron in different synchronous events. Middel panel: *CV*_*WithinEvent*_ (see Section 2.5) as a function of $\tau_{d}^{I}$, which quantifies the burstiness/regularity of pieces of spike trains within synchronous events. Right panel: *CV*_*events*_ (see Section 2.5) as a function of $\tau_{d}^{I}$, which quantifies the burstiness/regularity of the occurrence of synchronous events. **(D)** Index of HCC (see Section 2.5) as a function of $\tau_{d}^{I}$. The left panel of **(C)** is plotted from one simulation trial, while the other panels are plotted from averaging over 32 simulation trials. Error bars represent s.e.m.

When $\tau_{d}^{I}\geq 7\text{ ms}$, the LIF network works in synchronous states, and gradually goes from weak synchronous state to synchronously bursting state with the increase of $\tau_{d}^{I}$ (Figure 3A). The spike pattern structure in synchronous states is more complex than that in asynchronous states, and some key points are listed below:

1. *Synchronous firing*. Both the strength *p* and the duration τ_*cross*_ of synchronous events increase with $\tau_{d}^{I}$ (Figure 7A).
2. *Heterogeneity of rates*. The skewness and variance of the firing rates decrease with $\tau_{d}^{I}$ (Figure 7B).
3. *Auto-correlation structure*. For AT_SpikeNum_, because of heterogeneity of rates, different neurons fire quite different number of spikes in a synchronous event; but the spike number distribution for a neuron in different synchronous events is not broad (Figure 7C, left panel). For AT_WithinEvent_, if a neuron is to fire more than one spikes in a synchronous event, then these spikes tends to appear regularly (Figure 7C, middle panel): this is because that the refractory periods of the excitatory neurons in our model is fixed at $\tau_{ref}^{E}=2\text{ ms}$, so the neurons will fire regularly at rate close to $1∕\tau_{ref}^{E}$ if they receive strong excitatory inputs during synchronous events. For AT_events_, synchronous events in our model tends to appear regularly (Figure 7C, right panel), which reflects the oscillation nature of the E-I circuit (Brunel, 2000; Brunel and Wang, 2003).
4. *Heterogeneity of cross-correlations*. We found that the heterogeneity of cross-correlations tends to increase with $\tau_{d}^{I}$ (Figure 7D), especially when $\tau_{d}^{I}\geq 12\text{ ms}$. The reason of this phenomenon and its influence onto the efficacy variability will be discussed in **Section 3.5**.

### 3.2. Overview on the strategy of implementing spike shuffling methods onto the spike patterns of the LIF network

In the following two subsections, we will explain in details how we investigate the influence of spike pattern structure onto efficacy variability by implementing a variety of spike shuffling methods onto the spike patterns generated by the LIF network. But first of all, we give an overview on our strategy.

We use different spike shuffling methods for the spike patterns in asynchronous and synchronous states, because of the sharp difference of their pattern structure. These methods are explained in details in Section 2.6, Figure 5, and Supplementary Movie. For all the recorded spike patterns, we first treat them using a shuffling method called Whole-train Swap (WTS), before implementing any other method for further studies. The reason is that our spike patterns are generated by an underlying network with a specific structure, so the statistics of the spike patterns may be correlated with the structure of the underlying network. For example, if neuron *a* connects to neuron *b* in a network N, then the cross-correlation between these two neurons in the spike pattern P generated by N is more likely to be strong; so if the network N undergoes STDP according to P, then the synapse *a*→*b* is more likely to be potentiated strongly. Now suppose that there is another network N′ with the exactly the same structure with N, except that the neuronal labels are different. In this case, N and N′ will generate spike patterns with exactly the same statistics. However, neuron *a* may not connect to neuron *b* in N′, so if N′ also undergoes STDP according to P, then the strong cross-correlation between neuron *a* and neuron *b* in pattern P will not contribute to the efficacy variability of N′. Therefore, the efficacy variability of a network not only depends on the spike pattern statistics, but also depends on the possible pattern-network coupling. By comparing the efficacy variability under the original spike pattern and that under the spike pattern treated by WTS, we can understand how strongly this pattern-network coupling may contribute to the efficacy variability. We found that in both asynchronous and synchronous spike patterns of the LIF network, this pattern-network coupling can only slightly increase the efficacy variability (see **Figures 10A,B**). This suggests that the efficacy variability under both asynchronous and synchronous spike patterns can be largely understood by the spike pattern statistics alone. For all the spike patterns used in the following subsections, we first implement WTS to remove this pattern-network coupling.

From Figure 5, we see that a shuffling method may simultaneously destroy more than one aspects of pattern structure. Therefore, we must carefully design the order to implement them when trying to understand how each aspect of pattern structure influences efficacy variability. For example, to understand the effect of population rate fluctuation with time in asynchronous patterns, we would like to treat the patterns using TM. However, from Figure 5, TM destroys not only synchronous firing but also heterogeneity of cross-correlations, so to investigate the effect of synchronous firing, we must implement TM onto the spike patterns whose heterogeneity of cross-correlations are already destroyed. Therefore, we first treat the spike patterns using SS, thereby destroying heterogeneity of cross-correlations, and then investigate the change of the efficacy variability after further implementing TM. This is the basic idea we used to design our research.

We outline all the spike patterns we compare and the main results we obtained from the following sections in Table 1. For the convenience of the following discussions, we denote *P*_*S*_1_+*S*_2_+⋯_ to be the spike patterns sequentially shuffled by methods *S*_1_, *S*_2_, … from the spike patterns firstly shuffled by WTS. We denote *P*_0_ to be the original spike patterns, and *P*_*WTS*_ to be the patterns shuffled by WTS.

**Table 1:** **The spike patterns that we compare to understand the influence onto efficacy variability by different spike pattern structures, and the outline of main results**.

	**Spike pattern structures**		**The spike patterns we compare**	**Main results**
Asynchronous states ($\tau_{d}^{I}\leq 6\text{ ms}$)		SF	*P_SS_* vs. *P*_*SS*+*TM*_ (Term 1 in Section 3.3)	SF increases DiffV, influences DriftV by changing P-D imbalance (see Equation 11).
		HCC	*P*_*STR*_ vs. *P*_*STR*+*TM*_ (Term 2 in Section 3.3)	HCC increases DriftV under P-D balance.
		AT	*P_TM_* vs. *P*_*TM*+*SS*_ (Term 3 in Section 3.3)	*CV* increases DiffV, does not influence DriftV.
		HR	*P*_*TM*+*SS*_ vs. *P*_*TM*+*NRC*_ (Term 4 in Section 3.3)	HR and P-D imbalance together induce DriftV.
Synchronous states ($\tau_{d}^{I}\geq 7\text{ ms}$)		SF	*P_SSiE_* vs. *P*_*SSiE*+*STR*_ (Term 1 in Section 3.4)	SF increases DiffV, influences DriftV by changing P-D imbalance.
		HR	*P*_*SSiE*_ vs. *P*_*SSiE*+*TSiE*_ (Term 2 in Section 3.4)	HR and P-D imbalance together induce DriftV.
	AT	AT_WithinEvent_	*P_TSiE_* vs. *P*_*TSiE*+*SSiE*_ (Term 3 in Section 3.4)	Burstiness in AT_WithinEvent_ increases DiffV, does not change P-D imbalance.
		AT_SpikeNum_	*P*_*TSiE*+*SSiE*_ vs. *P*_*TSiE*+*NRC*_ (Term 4 in Section 3.4)	Burstiness in AT_SpikeNum_ increases DiffV, does not change P-D imbalance.
		AT_events_	*P_SSiE_* vs. *P*_*SSiE*+*ETS*_ (Term 5 in Section 3.4)	Burstiness in AT_events_ increases DiffV, potentiates (depresses) synapses when *A*_*p*_ > *A*_*d*_ (*A*_*p*_ < *A*_*d*_).
		HCC	*P_WTS_* vs. *P_SSiE_* (Section 3.5.2)	HCC is the main reason of DriftV in synchronous bursting states.

*SF, synchronous firing; AT, auto-correlation structure; HR, heterogeneity of rates; HCC, heterogeneity of cross-correlations. We use P_S_1_+S_2_+⋯_ to represent the spike patterns sequentially shuffled by methods S_1_, S_2_, … from the spike patterns firstly shuffled by WTS, and use P_WTS_ to represent the patterns treated by WTS*.

### 3.3. The influence of spike pattern structure onto efficacy variability in asynchronous patterns

Here we explain in details how we investigate the influence of spike pattern structure onto efficacy variability by implementing spike shuffling methods onto the spike patterns of the LIF network working in asynchronous states (when $\tau_{d}^{I}\leq 6\text{ ms}$).

**Figure 8:**
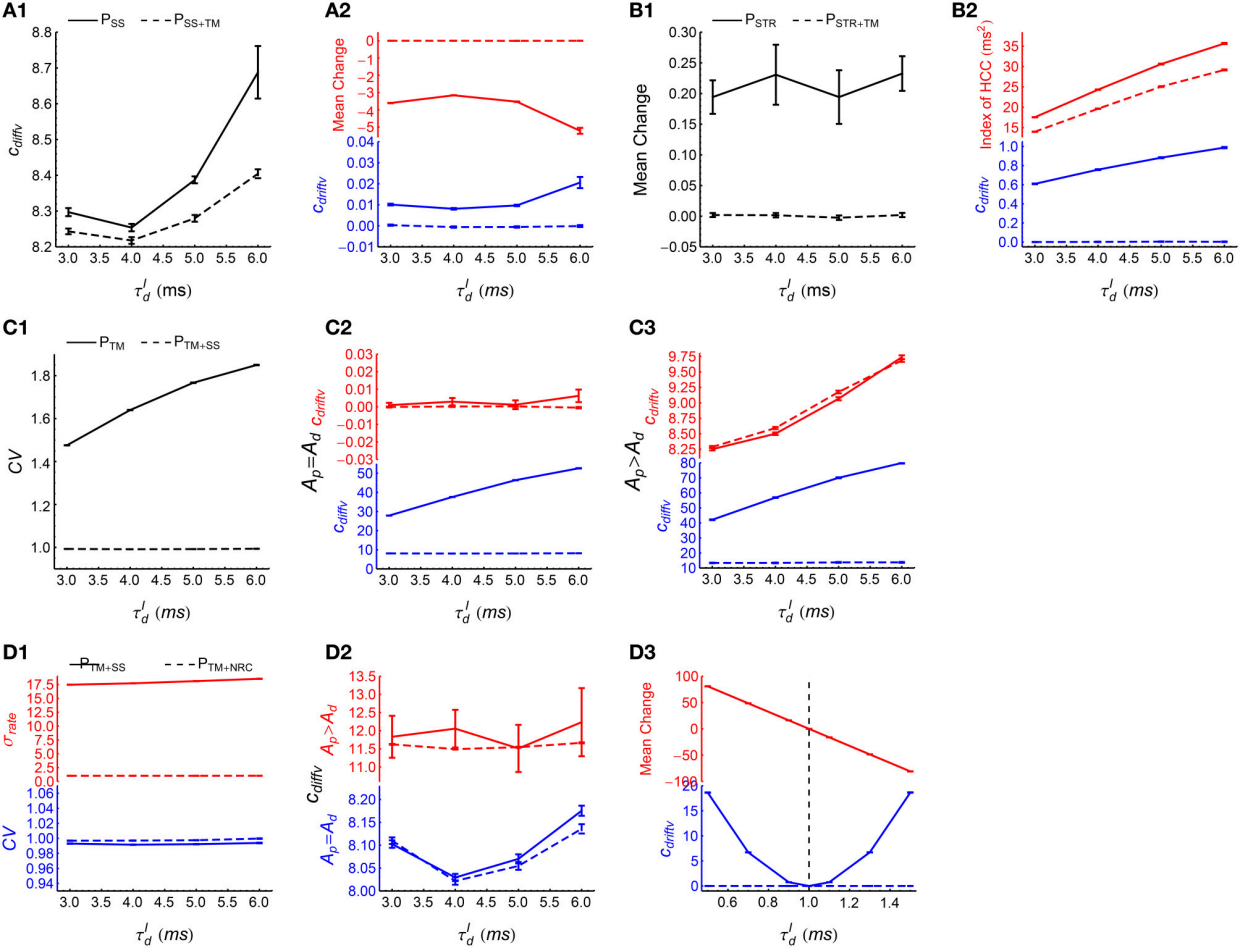
**How spike shuffling methods influence the efficacy variability and spike pattern statistics under asynchronous states**. **(A1,A2)** Comparing *P*_*SS*_ (solid line) with *P*_*SS*+*TM*_ (dashed line). **A1**: *c*_*diffv*_ is larger under *P*_*SS*_ than under *P*_*SS*+*TM*_. **A2:** *c*_*driftv*_ (blue) and ${\text{E}}_{\left(ab\right)}\left(\Delta {\tilde{w}}_{ab}\right)$ (red) are both nonzero under *P*_*SS*_, and are both almost zero under *P*_*SS*+*TM*_. Note that there are two panels marked in different colors in this subplot and some subplots below. Here, ${\text{E}}_{\left(ab\right)}\left(\Delta {\tilde{w}}_{ab}\right)$ represents the mean synaptic changes only caused by STDP (i.e., if the synaptic homeostasis is absent) (see Equation 11), and we use $\left|{\text{E}}_{\left(ab\right)}\left(\Delta {\tilde{w}}_{ab}\right)\right|$ to quantify P-D imbalance. The method for calculating *c*_*diffv*_ and *c*_*driftv*_ is explained in Section 2.4. **(B1,B2)** Comparing *P*_*STR*_ (solid line) with *P*_*STR*+*TM*_ (dashed line). **B1**: ${\text{E}}_{\left(ab\right)}\left(\Delta {\tilde{w}}_{ab}\right)$ is weak under *P*_*STR*_ and almost zero under *P*_*STR*+*TM*_. **B2**: *c*_*driftv*_ (blue) and the index of HCC (red) are both larger under *P*_*STR*_ than under *P*_*STR*+*TM*_. Here, the index of HCC quantifies the heterogeneity of cross-correlations (see Section 2.5), and the non-zero index of HCC under *P*_*STR*+*TM*_ manifests its background value caused by chance in the spike patterns. **(C1–C3)** Comparing *P*_*TM*_ (solid line) with *P*_*TM*+*SS*_ (dashed line). **C1**: The mean *CV* of spike trains under these two patterns. **C2**: *c*_*diffv*_ (blue) is larger under *P*_*TM*_ than under *P*_*TM*+*SS*_, but *c*_*driftv*_ (red) is almost the same. *A*_*p*_ = *A*_*d*_ = 1. **C3**: The same as **C2**, except that *A*_*p*_ = 4∕3 and *A*_*d*_ = 1. **(D1–D3)** Comparing *P*_*TM*+*SS*_ (solid line) with *P*_*TM*+*NRC*_ (dashed line). **D1**: Mean *CV* of spike trains (blue) and the standard deviation of firing rates σ_*rate*_ (red) under these two patterns. **D2**: *c*_*diffv*_ is almost the same under *P*_*TM*+*SS*_ with under *P*_*TM*+*NRC*_, both when *A*_*p*_ = *A*_*d*_ = 1 (blue), and when *A*_*p*_ = 4∕3 and *A*_*d*_ = 1 (red). **D3**: *c*_*driftv*_ (blue) and ${\text{E}}_{\left(ab\right)}\left(\Delta {\tilde{w}}_{ab}\right)$ (red) as functions of *A*_*d*_, keeping *A*_*p*_ = 1, $\tau_{d}^{I}=6\text{ ms}$. Note that under *P*_*TM*+*NRC*_, *c*_*driftv*_ ≈ 0; while under *P*_*TM*+*SS*_, *c*_*driftv*_ increases with $\left|{\text{E}}_{\left(ab\right)}\left(\Delta {\tilde{w}}_{ab}\right)\right|$, and *c*_*driftv*_ ≈ 0 only when $|{\text{E}}_{\left(ab\right)}\left(\Delta {\tilde{w}}_{ab}\right)|\approx 0$ (indicated by the vertical dashed line). In **(A1–D3)**, *A*_*p*_ = *A*_*d*_ = 1 by default. Simulations lasted for 41 s of biological time, and STDP and synaptic homeostasis started after 2 s of transient period. Error bars represent s.e.m. over 32 trials.

1. To examine the effect of synchronous firing, we observed the time evolution of the efficacy variability caused by *P*_*SS*_ and *P*_*SS*+*TM*_. From Figure 5, TM not only destroys synchronous firing, but also influences heterogeneity of cross-correlations, so we first destroy heterogeneity of cross-correlations using SS before implementing TM to study the effect of synchronous firing. As DiffV ∝ *t* and DriftV ∝ *t*^2^, we fit the time evolution of the efficacy variability with $c_{diffv}t+c_{driftv}t^{2}$ under *P*_*SS*_ and *P*_*SS*+*TM*_, with *c*_*diffv*_ and *c*_*driftv*_ being two to-be-fitted coefficients that respectively manifest the strengths of DiffV and DriftV. See Section 2.4 for fitting details. We found that *c*_*diffv*_ is larger under *P*_*SS*_ than under *P*_*SS*+*TM*_ (Figure 8A1), which manifests the contribution of synchronous firing onto DiffV. We also found that *c*_*driftv*_ is approximately zero under *P*_*SS*+*TM*_, but not (although weak) in *P*_*SS*_ (Figure 8A2, blue lower panel). To understand this, from Supplementary Materials Section S1.3.1, in the absence of heterogeneity of cross-correlations,
  (11) $$\begin{array}{r} \text{DriftV}\propto |{\text{E}}_{\left(ab\right)}\left(\Delta {\tilde{w}}_{ab}\right){|}^{2}{\text{Var}}_{a}\left(r_{a}\right), \end{array}$$
  with $\Delta {\tilde{w}}_{ab}$ being the change of the synapse from neuron *b* to neuron *a* only caused by STDP (without considering synaptic homeostasis), *E*_(*ab*)_ representing averaging over all the synapses, and Var_*a*_(*r*_*a*_) being the variance of the firing rates. In this equation, $\left|{\text{E}}_{\left(ab\right)}\left(\Delta {\tilde{w}}_{ab}\right)\right|$ quantifies the imbalance of the strengths of the potentiation and depression process under STDP (*P-D imbalance*), and we see that DriftV can be induced by the interaction of P-D imbalance and heterogeneity of rates. Because of the absence of synchronous firing in *P*_*SS*+*TM*_ and *A*_*p*_ = *A*_*d*_ in our model (Equation 5), the potentiation and depression of STDP are balanced in *P*_*SS*+*TM*_ (Figure 8A2, red upper panel), but not in *P*_*SS*_ because of the population rate fluctuation with time (Figure 6A, upper panels). This weak P-D imbalance under *P*_*SS*_ makes DriftV non-zero under heterogeneity of firing rates, which makes *c*_*driftv*_ non-zero. These findings suggest that in asynchronous spike patterns, the fluctuation of population rate increases DiffV, and influences DriftV under heterogeneity of rates by changing P-D imbalance.
2. To manifest the effect of heterogeneity of cross-correlations, we compared the efficacy variability under *P*_*STR*_ and *P*_*STR*+*TM*_. Here we use STR to flatten the population rate fluctuation with time, before further using TM to study the effect of heterogeneity of cross-correlations. We found that *c*_*driftv*_ is significantly larger than zero under *P*_*STR*_ but close to zero under *P*_*STR*+*TM*_ (Figure 8B2, blue lower panel). From Supplementary Materials Section S1.3.2, DriftV can be largely considered to be contributed by two factors: heterogeneity of cross-correlations and the interaction between P-D imbalance and heterogeneity of rates. To understand the non-zero DriftV in *P*_*STR*_, note that *P*_*STR*_ is free of population rate fluctuation, so because of *A*_*p*_ = *A*_*d*_ in our model (Equation 5), we should expect P-D *balance* in *P*_*STR*_. Although P-D *imbalance* may slightly exist in *P*_*STR*_ (Figure 8B1), it is fairly weak. To understand how strong DriftV this weak P-D imbalance contributes, we can compare the P-D imbalance and DriftV under *P*_*STR*_ with those under *P*_*SS*_ (Figure 8A2, blue lower panel and Figure 8B2, blue lower panel). We can see that the P-D imbalance under *P*_*SS*_ is much stronger than that under *P*_*STR*_, but the DriftV under *P*_*SS*_ is much weaker than that under *P*_*STR*_. These suggest that the strong DriftV under *P*_*STR*_ is not caused by the weak P-D imbalance, but instead by the heterogeneity of cross-correlations remnant from the original patterns (Figure 6D). TM destroys this heterogeneity of cross-correlations (Figure 8B), and *c*_*driftv*_ accordingly becomes almost zero (Figure 8B2, blue lower panel).
3. To examine the effect of auto-correlation structure, we compared the efficacy variability under *P*_*TM*_ with that under *P*_*TM*+*SS*_. In these two patterns, synchronous firing and heterogeneity of cross-correlations are destroyed by TM; and SS then is used to reduce *CV* to almost 1 (Figure 8C1). We studied both the case *A*_*p*_ = *A*_*d*_ and the case *A*_*p*_ ≠ *A*_*d*_ here to investigate whether the influence of auto-correlation structure onto DriftV depends on P-D imbalance. We found that SS hardly influences *c*_*driftv*_ but significantly reduces *c*_*diffv*_ (Figures 8C2,C3). This suggests that the burstiness of stationary spike trains does not change DriftV, but increases DiffV.
4. To study the effect of heterogeneity of rates, we compared the efficacy variability under *P*_*TM*+*SS*_ and *P*_*TM*+*NRC*_. Both SS and NRC destroy heterogeneity of cross-correlations, and result in spike trains with *CV* ≈ 1 (Figure 8D1, blue lower panel), except that SS keeps the heterogeneity of rates, while NRC homogenizes the firing rates (Figure 8D1, red upper panel). We found that *c*_*diffv*_ is almost the same under *P*_*TM*+*SS*_ and *P*_*TM*+*NRC*_, regardless whether *A*_*p*_ = *A*_*d*_ or *A*_*p*_ ≠ *A*_*d*_ (Figure 8D2), which suggests that heterogeneity of rates does not significantly change DiffV if the *CV* of the spike trains is kept around 1. To understand the influence of heterogeneity of rates onto DriftV, we kept *A*_*p*_ = 1, and observed how *c*_*driftv*_ and P-D imbalance change with *A*_*d*_ under these two patterns. We found that *c*_*driftv*_ is always near to zero under *P*_*TM*+*NRC*_, but is positively correlated with the strength of P-D imbalance under *P*_*TM*+*SS*_ (Figure 8D3). This shows that P-D imbalance can induce DriftV under heterogeneity of rates.

### 3.4. The influence of spike pattern structure onto efficacy variability in synchronous patterns

Here we explain in details how we investigate the influence of spike pattern structure onto efficacy variability by implementing spike shuffling methods onto the spike patterns of the LIF network working in synchronous states (when $\tau_{d}^{I}\geq 7\text{ ms}$).

**Figure 9:**
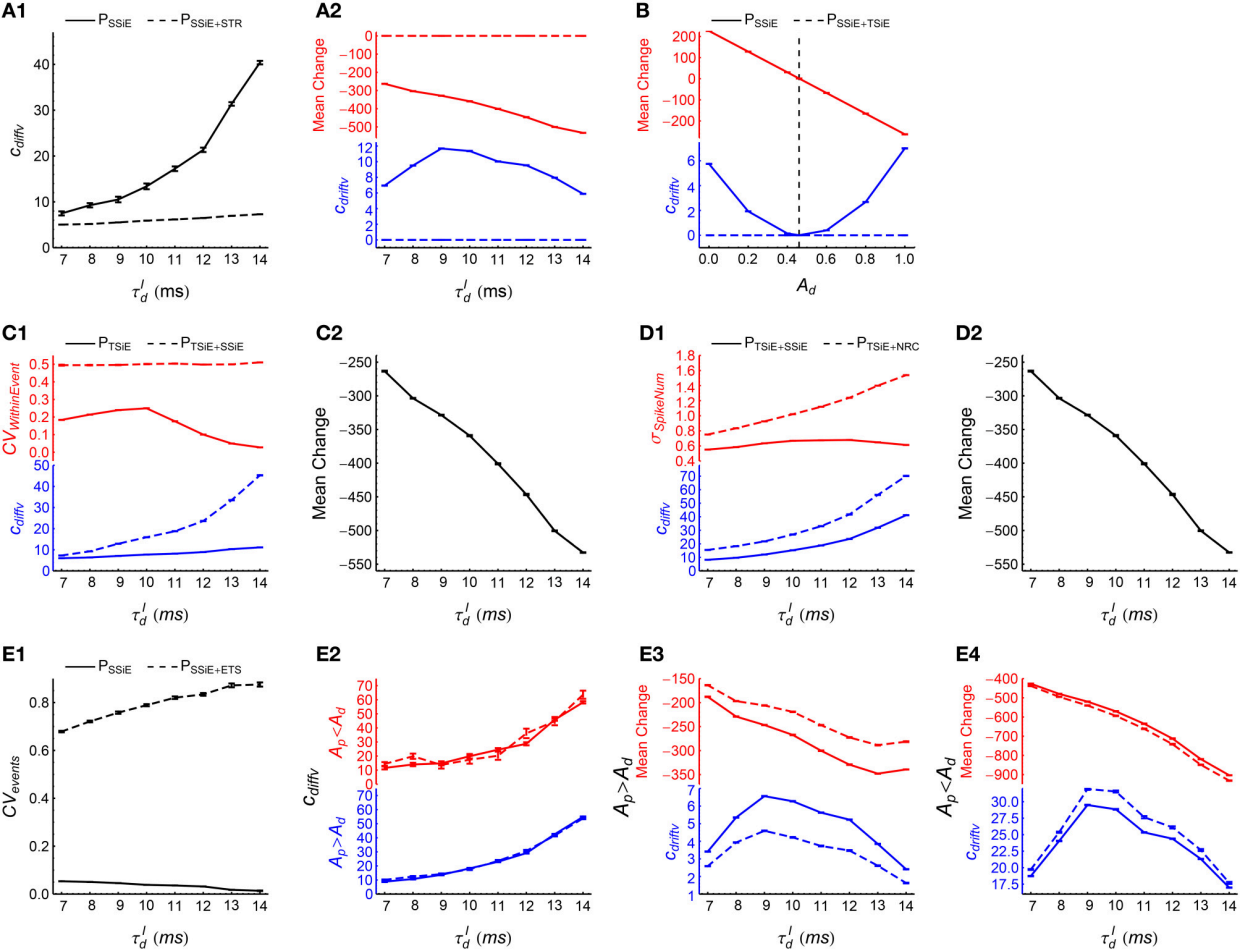
**How spike shuffling methods influence the efficacy variability and spike pattern statistics under synchronous states**. **(A1,A2)** Comparing *P*_*SSiE*_ (solid line) with *P*_*SSiE*+*STR*_ (dashed line). **A1**: *c*_*diffv*_ is larger under *P*_*SSiE*_ than under *P*_*SSiE*+*STR*_. **A2:** *c*_*driftv*_ (blue) and ${\text{E}}_{\left(ab\right)}\left(\Delta {\tilde{w}}_{ab}\right)$ (red) are both nonzero under *P*_*SS*_, and are both almost zero under *P*_*SS*+*STR*_. Note that there are two panels marked in different colors in this subplot and some subplots below. See the caption of Figure 8A2 for the meaning of ${\text{E}}_{\left(ab\right)}\left(\Delta {\tilde{w}}_{ab}\right)$. **(B)** Comparing *P*_*SSiE*_ (solid line) with *P*_*SSiE*+*TSiE*_ (dashed line). *c*_*driftv*_ and ${\text{E}}_{\left(ab\right)}\left(\Delta {\tilde{w}}_{ab}\right)$ as functions of *A*_*d*_, keeping *A*_*p*_ = 1 and $\tau_{d}^{I}=7\text{ ms}$. Note that under *P*_*SSiE*+*TSiE*_, *c*_*driftv*_ ≈ 0; while under *P*_*SSiE*_, *c*_*driftv*_ increases with $\left|{\text{E}}_{\left(ab\right)}\left(\Delta {\tilde{w}}_{ab}\right)\right|$, and *c*_*driftv*_ ≈ 0 only when $|{\text{E}}_{\left(ab\right)}\left(\Delta {\tilde{w}}_{ab}\right)|\approx 0$ (indicated by the vertical dashed line). **(C1,C2)** Comparing *P*_*TSiE*+*SSiE*_ (solid line) with *P*_*TSiE*+*NRC*_ (dashed line). **C1**: Both *c*_*diffv*_ and σ_*SpikeNum*_ are larger under *P*_*TSiE*+*NRC*_ than under *P*_*TSiE*+*SSiE*_. Here, σ_*SpikeNum*_ represents the standard deviation of the distribution of spike number per neuron per synchronous event. **C2**: ${\text{E}}_{\left(ab\right)}\left(\Delta {\tilde{w}}_{ab}\right)$ under these two patterns strongly overlap. **(D1,D2)** Comparing *P*_*TSiE*_ (solid line) with *P*_*TSiE*+*SSiE*_ (dashed line). **D1**: Both *c*_*diffv*_ and *CV*_*WithinEvent*_ are larger under *P*_*TSiE*_ than under *P*_*TSiE*+*SSiE*_. Here, *CV*_*WithinEvents*_ quantifies burstiness/regularity of the pieces of spike trains within synchronous events (See Section 2.5 for details). **D2**: ${\text{E}}_{\left(ab\right)}\left(\Delta {\tilde{w}}_{ab}\right)$ under these two patterns strongly overlap. **(E1–E4)** Comparing *P*_*TSiE*_ (solid line) and *P*_*TSiE*+*ETS*_ (dashed line). **E1**: The burstiness of the occurrence of synchronous events (quantified by *CV*_*events*_, see Section 2.5) under these two patterns. **E2**: *c*_*diffv*_ under these two patterns both when *A*_*p*_ = 4.∕3 and *A*_*d*_ = 1 (blue), and when *A*_*p*_ = 1 and *A*_*d*_ = 4.∕3 (red). **E3**: ${\text{E}}_{\left(ab\right)}\left(\Delta {\tilde{w}}_{ab}\right)$ (red) and *c*_*driftv*_ (blue) under these two patterns when *A*_*p*_ = 4.∕3 and *A*_*d*_ = 1. **E4**: The same as **E3**, except that *A*_*p*_ = 1 and *A*_*d*_ = 4.∕3. In **(A1–E4)**, *A*_*p*_ = *A*_*d*_ = 1 by default. Simulations lasted for 41 s of biological time, and STDP and synaptic homeostasis started after 2 s of transient period. Error bars represent s.e.m. over 32 trials.

1. To examine the effects of synchronous firing, we fitted the time evolution of efficacy variability with $c_{diffv}t+c_{driftv}t^{2}$ under *P*_*SSiE*_ and *P*_*SSiE*+*STR*_. Here we would like to use STR to flatten the population firing rate. But as STR not only destroys synchronous firing, but also influences auto-correlation structure and heterogeneity of cross-correlations (Figure 5), we first implement SSiE to result in inhomogeneous Poisson processes before investigating the effect of synchronous firing using STR. We found that *c*_*diffv*_ under *P*_*SSiE*_ is larger than that under *P*_*SSiE*+*STR*_ (Figure 9A1), which manifests the contribution of synchronous firing onto DiffV. We also found that *c*_*driftv*_ is approximately zero under *P*_*SSiE*+*STR*_, but not in *P*_*SSiE*_ (Figure 9A2, blue lower panel). The reason is that the potentiation and depression of STDP are balanced in *P*_*SSiE*+*STR*_ , because of the absence of synchronous firing in *P*_*SSiE*+*STR*_ and *A*_*p*_ = *A*_*d*_ in our model (Equation 8); but not in *P*_*SSiE*_, because of the population rate fluctuation with time (Figure 9A2, red upper panel). And this P-D imbalance results in DriftV under heterogeneity of rates. These findings suggest that synchronous firing generally increases DiffV, and influences DriftV under heterogeneity of rates by changing P-D imbalance, which are consistent with our results for asynchronous patterns (see Term 1 in the previous section).
2. To examine the effects of heterogeneity of rates, we compared the efficacy variability under *P*_*SSiE*_ with that under *P*_*SSiE*+*TSiE*_. SSiE destroys heterogeneity of cross-correlations, so that the interaction of heterogeneity of rates and P-D imbalance becomes the only possible source of DriftV. TSiE further destroys heterogeneity of rates, which reduces the DriftV (i.e., *c*_*driftv*_) under *P*_*SSiE*+*TSiE*_ almost to zero (Figure 9B, blue lower panel). When we keep *A*_*p*_ = 1 but assign *A*_*d*_ different values, the P-D imbalance is accordingly changed. We found that *c*_*driftv*_ under *P*_*SSiE*_ is positively correlated with the strength of P-D imbalance (Figure 9B). These observations again support the idea that P-D imbalance can induce DriftV under heterogeneity of rates (see Term 4 in the previous section).
3. To examine the effects of AT_WithinEvent_, we compared the efficacy variability under *P*_*TSiE*_ with that under *P*_*TSiE*+*SSiE*_. By definition (Figure 5), SSiE not only randomizes the spike times, but also destroys the heterogeneity of cross-correlations. So before using SSiE to investigate the effect of AT_WithinEvent_, we first implemented TSiE to destroy heterogeneity of cross-correlations in these two patterns. We found that after SSiE, the pieces of spike trains within synchronous events becomes burstier (Figure 9C1, red upper panel), and *c*_*diffv*_ also becomes larger (Figure 9C1, blue lower panel). This suggests that burstier spike trains within synchronous events increases DiffV.
  In these two patterns, TSiE destroys both heterogeneity of cross-correlations and heterogeneity of rates (which are the two sources of DriftV), so *c*_*driftv*_ ≈ 0. To understand the possible influence of AT_WithinEvent_ onto DriftV under heterogeneity of rates, we compared the P-D imbalance under *P*_*TSiE*_ with that under *P*_*TSiE*+*SSiE*_. We found that the P-D imbalance under these two patterns are almost the same (Figure 9C2), which suggests that AT_WithinEvent_ has no influence onto DriftV under heterogeneity of rates.
4. To examine the effects of AT_SpikeNum_, we compared the efficacy variability under *P*_*TSiE*+*SSiE*_ with that under *P*_*TSiE*+*NRC*_. In these two patterns, TSiE homogenizes firing rates, so that different neurons have almost the same spike number distribution across synchronous events. Both SSiE and NRC randomize spike times (so that *P*_*TSiE*+*SSiE*_ and *P*_*TSiE*+*NRC*_ have similar AT_WithinEvent_, see Supplementary Materials Section S4 for more discussions); but SSiE keeps this spike number distribution, while NRC makes this distribution broader (Figure 9D1, red upper panel). We found that *c*_*diffv*_ is larger under *P*_*TSiE*+*NRC*_ than under *P*_*TSiE*+*SSiE*_ (Figure 9D1, blue lower panel). This suggests that broader distribution of the spike numbers a neuron fires in different synchronous events increases DiffV.
  We also found that P-D imbalance under these two patterns are almost the same (Figure 9D2), which suggests that AT_SpikeNum_ has no influence onto DriftV under heterogeneity of rates.
5. To examine the effects of AT_events_, we compared the efficacy variability under *P*_*SSiE*_ with that under *P*_*SSiE*+*ETS*_. As ETS changes not only AT_events_ but also heterogeneity of cross-correlations (Figure 5), we first implemented SSiE to destroy heterogeneity of cross-correlations before further using ETS to study the effects of AT_events_. ETS increases the burstiness of the occurrence of synchronous events (Figure 9E1), but we found that *c*_*diffv*_ is almost the same under these two patterns (Figure 9E2). However, this is possibly because that the irregularity of spike trains within synchronous events dominates DiffV, while the contribution from the interactions between spikes in different synchronous events are small due to the far separation between synchronous events and the decaying STDP time window. In Supplementary Material Section S5.1.4, we study the influence of *CV*_*events*_ onto DiffV in more details by comparing *P*_*TSiE*+*SSiE*_ with *P*_*TSiE*+*SSiE*+*ETS*_, with TSiE being used to homogenize firing rate to make DriftV = 0. This makes the efficacy variability totally caused by DiffV, so that the estimated value of *c*_*diffv*_ becomes more precise. To increase the STDP interaction between spikes in different synchronous events, we also increase the STDP time scale τ_*STDP*_ (see Equation 5). We find that *c*_*diffv*_ under *P*_*TSiE*+*SSiE*+*ETS*_ is larger than that under *P*_*TSiE*+*SSiE*_ (Supplementary Figure 6), which suggests that burstier occurrence of synchronous events tends to increase DiffV.
  How AT_events_ influences DriftV may be intriguing. Our previous paper (Bi and Zhou, 2016) suggests that if synchronous-event overlapping is absent (i.e., if a synchronous event S happens during the interval [*t*_1_, *t*_2_], then no other synchronous event should happen within [*t*_1_ − τ_*delay*_, *t*_2_], with τ_*delay*_ being the axonal delay), then the burstiness of the occurrence of synchronous events tends to potentiate synapses when *A*_*p*_ > *A*_*d*_ and depress synapses when *A*_*p*_ < *A*_*d*_. We carefully avoid synchronous-event overlapping during ETS (see Section 2.6.3.3). Consistently, we found that ETS tends to potentiate synapses when *A*_*p*_ > *A*_*d*_ and depress synapses when *A*_*p*_ < *A*_*d*_ (Figures 9E3,E4, red upper panels), which is consistent with our previous results. In our model, this weakens (or strengthens) P-D imbalance (i.e., the absolute value of mean synaptic changes under STDP) when *A*_*p*_ > *A*_*d*_ (or *A*_*p*_ < *A*_*d*_), which decreases (or increases) DriftV under heterogeneity of rates (Figures 9E3,E4, blue lower panels).

### 3.5. Understanding the efficacy variability under the spike patterns generated by the LIF network

The influence of synaptic time scales onto network dynamics has been discussed in theoretical studies (Brunel and Wang, 2003; Geisler et al., 2005). When the decay time scale of inhibitory conductance $\tau_{d}^{I}\leq 6\text{ ms}$, our LIF network works in asynchronous state with the firing rate of each neuron fluctuating chaotically (Figure 6A). It is believed that the high-dimensional nature of the state trajectory of such networks provides substrate for complex computations such as classifying temporal signals (Ostojic, 2014) and learning complex spatio-temporal patterns (Sussillo and Abbott, 2009), also see the seminal papers by Maass et al. (2002) and Jaeger (2003) on reservoir computing. When $\tau_{d}^{I}=7$ or 8 ms, the network works in weak synchronous state (Figure 3A, middle panel). In this case, the oscillation frequency is larger than the firing frequency of most neurons, and the spike trains of most neurons look irregular despite the population coherent rhythm, which is consistent with the observations in hippocampus and cortex (Csicsvari et al., 1998; Fries et al., 2001). When $\tau_{d}^{I}$ continues to grow (especially when $\tau_{d}^{I}=13\text{ ms}$ or 14 ms), nearly every neuron fires burstly in a synchronous event (Figure 3A, right panel), just like in the spike patterns observed in epilepsy (Gulyás and Freund, 2015). In the following discussions, we will use spike shuffling methods to understand the contributions of different statistical features to the efficacy variability under these spike patterns generated by our LIF network, thereby gaining understanding on the efficacy variability under the spike patterns observed in these theoretical and experimental studies. For example, we would like to know which spike pattern statistics influences to the efficacy variability significantly, and which spike pattern statistics is the main reason for the change of efficacy variability with $\tau_{d}^{I}$. In our simulations, we kept the firing rate of the excitatory population at 20 Hz for different $\tau_{d}^{I}$s (see Section 2.3), so that the change of the efficacy variability with $\tau_{d}^{I}$ is totally caused by higher order pattern statistics.

#### 3.5.1. Asynchronous states

**Figure 10:**
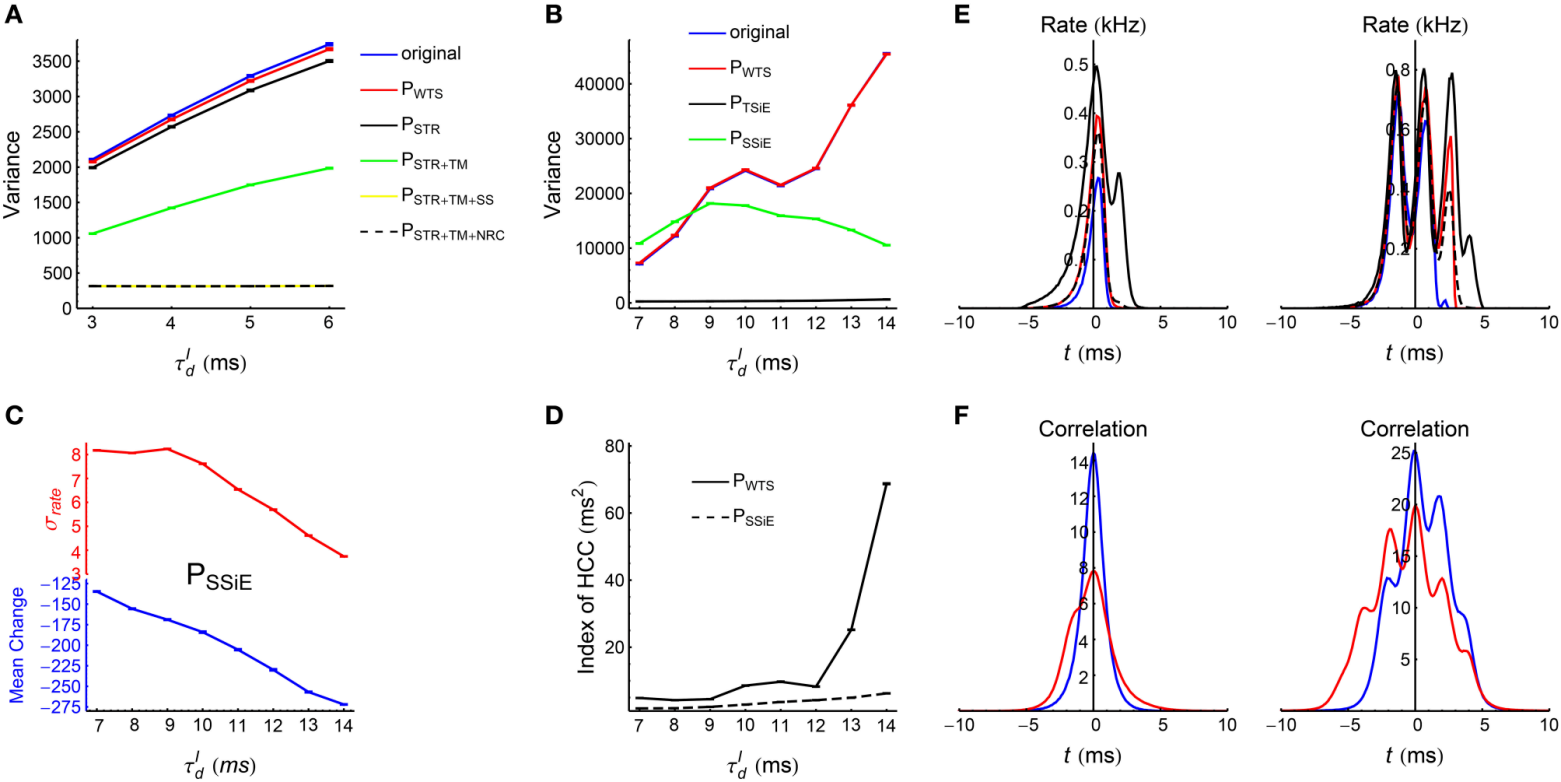
**Understanding the contributions to the efficacy variability by different spike pattern structures under the spike patterns of the LIF network**. **(A)** The efficacy variance under the original asynchronous patterns and the patterns sequentially shuffled by WTS, STR, TM, SS, and NRC. Note that the variance under *P*_*STR*+*TM*+*SS*_ and that under *P*_*STR*+*TM*+*NRC*_ are strongly overlapped, because of the P-D *balance* caused by the absence of population rate fluctuation after STR and *A*_*p*_ = *A*_*d*_ in our model. **(B)** The efficacy variance under the original synchronous spike patterns and under *P*_*WTS*_, *P*_*TSiE*_ and *P*_*SSiE*_. Note that the results for the original pattern and *P*_*WTS*_ are strongly overlapped. **(C)** ${\text{E}}_{\left(ab\right)}\left(\Delta {\tilde{w}}_{ab}\right)$ (blue) and the standard deviation of firing rate σ_*rate*_ (red) as functions of $\tau_{d}^{I}$ under *P*_*SSiE*_. **(D)** Index of HCC in *P*_*WTS*_ and *P*_*SSiE*_. **(E)** Firing profiles within synchronous events under *P*_*WTS*_ for neurons with high (blue), middle (red) and low (black) firing rates as well as the whole population (dashed), when $\tau_{d}^{I}=7$ ms (left panel) and 14 ms (right panel). The zero point indicates the middle time of a synchronous event, and the function value at time *t* means the firing rate at time *t* relative to the middle time of the synchronous event, averaging over all synchronous events in the spike patterns. See Section 2.5 for details. **(F)** The unit cross-correlation between the neurons with low rate and middle rate (blue), and that between the neurons with high rate and middle rate (red), when $\tau_{d}^{I}=7$ ms (left panel) and 14 ms (right panel). The definitions of “high rate,” “middle rate,” and “low rate” neurons are the same as those in **(E)** (see Section 2.5 for details). In **(A–F)**, *A*_*p*_ = *A*_*d*_ = 1. Simulations lasted for 41 s of biological time, and STDP and synaptic homeostasis started after 2 s of transient period. Error bars represent s.e.m. over 32 trials.

For asynchronous states, we sequentially shuffled the original spike pattern by WTS, STR, TM, SS, and NRC, and then observed the efficacy variability in the resulting spike patterns (Figure 10A). The change of the efficacy variability after each of these shuffling methods respectively manifests the contribution of pattern-network coupling (for WTS), population rate fluctuation with time (for STR), heterogeneity of cross-correlations (for TM), auto-correlation structure (for SS), and heterogeneity of rates (for NRC). Here, after WTS, we first use STR to assess the influence of population rate fluctuation onto efficacy variability. From Figure 5, STR not only flattens population rate, but also influence auto-correlation structure and heterogeneity of cross-correlations. However, as the fluctuation of population rate in asynchronous states is not so strong, the auto-correlation structure and heterogeneity of cross-correlation in the original patterns should largely preserve after STR. From Figure 10A, the efficacy variability is only slightly reduced by STR. After STR, both *CV* and index of heterogeneity of cross-correlation are slightly reduced (Supplementary Figure 8), both of which reduce the efficacy variability. This suggests that the reduction of the efficacy variability caused by population rate fluctuation alone is even smaller than the reduction by STR shown in Figure 10A. Afterwards, we implement TM onto patterns treated by STR for understanding the contribution of heterogeneity of cross-correlations, implement SS onto patterns treated by TM for auto-correlation structure, and compare the effects of SS and NRC on patterns treated by TM for heterogeneity of rates. All of these are consistent with the research design in the previous subsections (see the items about asynchronous states in Table 1).

We found that under our parameter values, heterogeneity of cross-correlations and auto-correlation structure are the two major sources of the efficacy variability; and auto-correlation structure contributes most to the increase of the efficacy variability with $\tau_{d}^{I}$ (Figure 10A). As heterogeneity of cross-correlations mainly contributes to DriftV (which is of O(*t*^2^) order), and auto-correlation structure mainly contributes to DiffV (which is of only O(*t*) order), the significant contribution of auto-correlation structure here highlights its importance to understand the efficacy variability in asynchronous states.

#### 3.5.2. Synchronous states

For synchronous states, we first used WTS to destroy the pattern-network coupling, and found that this coupling hardly influences efficacy variability (Figure 10B). Then, we used TSiE to destroy heterogeneity of rates and heterogeneity of cross-correlations, which are the two sources of DriftV, and found that the efficacy variability is reduced by more than 10 times after TSiE (Figure 10B). This suggests that the efficacy variability under the synchronous states in our model is dominated by DriftV, and we only need to focus on DriftV to understand the change of the efficacy variability with $\tau_{d}^{I}$.

To compare the contribution of heterogeneity of rates and heterogeneity of cross-correlation to DriftV, we compared the efficacy variability under *P*_*WTS*_ with that under *P*_*SSiE*_ (Figure 10B). After SSiE, the heterogeneity of cross-correlations is destroyed. Therefore, the DriftV under *P*_*SSiE*_ is contributed by the interaction of heterogeneity of rates and P-D imbalance. We found that the efficacy variability under *P*_*SSiE*_ gets its maximum around $\tau_{d}^{I}=9\text{ ms}$, and decreases when $\tau_{d}^{I}$ continues to grow (Figure 10B). This can be understood as a result caused by the interaction of the increase of P-D imbalance and the decrease of rate heterogeneity with $\tau_{d}^{I}$ (Figure 10C). However, the efficacy variability under *P*_*WTS*_ is smaller than that under *P*_*SSiE*_ when $\tau_{d}^{I}$ is small, but tends to monotonically increase with $\tau_{d}^{I}$ (Figure 10B), which manifests the contribution of heterogeneity of cross-correlations. Indeed, we found that the index of HCC (which quantifies ${\text{Var}}_{a}\left(\int_{-\infty }^{\infty }\text{d}\tau H\left(\tau \right)C_{a}\left(-\tau_{delay}-\tau \right)\right)$, see Section 2.5) tends to increase with $\tau_{d}^{I}$, especially in the strong-bursting regime (i.e., $\tau_{d}^{I}=13\text{ ms},14\text{ ms}$, see Figure 10D). Together, these observations make us come to the understanding that: (1) in weak synchronous states (when $\tau_{d}^{I}$ is small), heterogeneity of rates contributes most to the DriftV, and heterogeneity of cross-correlations reduces the DriftV caused by heterogeneity of rates; (2) in synchronous bursting states (when $\tau_{d}^{I}$ is large), heterogeneity of cross-correlation overwhelms heterogeneity of rates to be the dominating factor to DriftV, which pushes DriftV to continuously increase with τ_*d, I*_.

To understand the origin of the heterogeneity of cross-correlations in synchronous states, we plot the firing profiles of the neurons during synchronous events (Figure 10E). We found that under *P*_*WTS*_, in weak synchronous states (when $\tau_{d}^{I}$ is small), the neurons with high firing rates (*high-rate neurons*) tends to start to fire earlier and stop to fire later than the neurons with low firing rates (*low-rate neurons*) in a synchronous event (Figure 10E, left panels); in synchronous bursting states (when $\tau_{d}^{I}$ is large), however, high-rate neurons still stop to fire later than low-rate neurons at the end of a synchronous event, but high-rate and low-rate neurons tend to start to fire at the same time with the same rate at the beginning of a synchronous event (Figure 10E, right panels). To understand this, note that the high-rate (low-rate) neurons tend to receive more (less) excitatory inputs and less (more) inhibitory inputs in the network, which makes the inputs of high-rate neurons increase above (decrease below) the firing threshold earlier (later) than those of the low-rate neurons at the beginning (end) of a synchronous event. This is the reason for the firing profiles when $\tau_{d}^{I}$ is smaller. When $\tau_{d}^{I}$ is large, however, the strengths of the inhibitory currents in our model (Equation 8) are accordingly scaled down. This enlarges the time window for the supra-threshold excitatory currents to quickly push the firing rates of all the neurons to near saturation at the beginning of a synchronous event, before the time-integrations of the inhibitory currents gradually turn off the neuronal activities, from low-rate to high-rate neurons. Now suppose that the 0th neuron in the network receives both from the *h*th neuron with higher firing rate and from the *l*th neuron with lower firing rate. When $\tau_{d}^{I}$ is small, both the unit cross-correlation between the *h*th and 0th neurons *C*_*h*0_(τ) and that between the *l*th and 0th neurons *C*_*l*0_(τ) are almost symmetric around τ = 0 (Figure 10F, left panel). But when $\tau_{d}^{I}$ is large, there is an apparent left-shift of *C*_*h*0_(τ) comparing with *C*_*l*0_(τ) (Figure 10F, right panel): this causes $\int_{-\infty }^{\infty }\text{d}\tau H\left(\tau \right)C_{h0}\left(-\tau_{delay}-\tau \right)$ very different from $\int_{-\infty }^{\infty }\text{d}\tau H\left(\tau \right)C_{l0}\left(-\tau_{delay}-\tau \right)$ when $\tau_{d}^{I}$ is large. This is the reason for the increase of heterogeneity of cross-correlations with $\tau_{d}^{I}$.

Another issue that has not been considered till now is the possible dependence (on, say, spike times or spike numbers) between the pieces of spike trains in adjacent synchronous events. To check the influence of this inter-event dependence onto the efficacy variability, we compared the efficacy variability under *P*_*WTS*_ with that under *P*_*EOS*_ (see Section 2.6.3.4 for the method of EOS). We found that EOS hardly influences the efficacy variability (Supplementary Figure 9), which suggests that this inter-event dependence hardly influences the efficacy variability in our model.

## 4. Discussion

In this paper, we developed a systematic spike shuffling approach to alter four types of statistics of spike patterns (i.e., synchronous firing, auto-correlation structure, heterogeneity of rates and heterogeneity of cross-correlations) under both asynchronous and synchronous states, and then applied this approach to systematically study the influence of the four aspects of pattern structures onto the efficacy variability under STDP and synaptic homeostasis in the spike patterns self-organized by a biologically plausible LIF neuronal network. The main results are shown in Table 1, which can be summarized as (1) synchronous firing and burstiness tend to increase DiffV, (2) synchronous firing influences P-D imbalance, which can induce DriftV together with heterogeneity of rates, and (3) heterogeneity of cross-correlations induces DriftV together with heterogeneity of rates. We compared our results with those of our previous paper (Bi and Zhou, 2016) in Supplementary Material Section S5, and found their consistency. We also examined the contributions of different pattern statistics to the efficacy variability under the spike patterns of the LIF network, and found that auto-correlation structure is important to determine the efficacy variability under asynchronous states, while heterogeneity of cross-correlations is the main factor to cause efficacy variability when the network moves into synchronous bursting states. We believe our method can contribute to the library of spike shuffling methods, and provide new angles for experimentalists to analyze their data; and our results can help to understand the efficacy variability under the spike patterns observed in theoretical and experimental studies.

When the time scales of inhibition and excitation are comparable, our LIF network works in asynchronous states; when the inhibition time scale starts to grow, the LIF network transites to weak synchronous state at some point, performing oscillation in the gamma frequency regime (Figure 3A). In our simulations, we fixed the excitatory decay time constant at 4 ms while changed the inhibitory decay time constant within 3–14 ms, both are around typical values of AMPA and GABA_A_ currents [2 ~ 5 ms for AMPA (Zhou and Hablitz, 1998; Angulo et al., 1999), 5 ~ 15 ms for GABA_A_ (Xiang et al., 1998; Gupta et al., 2000)]. In physiological situations, neuronal network dynamics is not only determined by synaptic time scales, but also by other factors such as the neuronal response properties and connections; so it is difficult to directly compare the spike patterns in our simulations with those observed in experiments. However, we can still gain insight onto the functional roles of the efficacy variability caused by the dynamics through the computational tasks that the network takes.

In the asynchronous states, the firing rate of each neuron in our LIF network fluctuates chaotically (Figure 6A, lower panels), and our study suggests that the irregular auto-correlation structure of spike trains in this chaotic asynchronous state may contribute significantly to the efficacy variability of the recurrent connections. Theoretically, it has been proposed that larger variance of recurrent synaptic weights facilitates chaoticity of a network (Sompolinsky et al., 1988; Toyoizumi and Abbott, 2011), which again promotes irregularity of spike trains. This mutual facilitation of chaos and synaptic variance may have important implications in the development of brain areas such as the primary olfactory system and the cerebellum, which may use the high-dimensional nature of the state trajectory in chaos to do complex computations such as odor discrimination (Mazor and Laurent, 2005) and motion control (Buonomano and Mauk, 1994; Yamazaki and Tanaka, 2007). One problem of such chaos-based computations is that the dynamics may be sensitive to the noises and the initial conditions of the network. This sensitivity may be suppressed using a feedback loop from the readout unit (Jaeger and Haas, 2004; Sussillo and Abbott, 2009) or perform suitable plastic changes on the recurrent weights (Laje and Buonomano, 2013), so that the innate trajectory becomes stabilized.

Gamma oscillations is believed important for memory formation under normal physiological conditions (Sederberg et al., 2007; Jutras et al., 2009; Yamamoto et al., 2014). Our intuition shown in Figure 2A suggests that successful memory embedding requires small efficacy variability, which may be a physiological function of gamma oscillation. However, in our simulations, we did not observe small efficacy variability when our LIF network works in weak synchronous state (Supplementary Figure 7). This may be because that we did not consider the shunting effect of perisomatic fast-spiking interneurons in our model. On the one hand, these interneurons may help to homogenize neuronal firing rates (Vida et al., 2006), which reduces DriftV. On the other hand, they are also able to entrain high temporal precision of spike time (Cobb et al., 1995; Salkoff et al., 2015), resulting in spike-to-spike synchrony of pyramidal neurons, which helps to reduce DiffV (see Section 3.3 of our previous paper Bi and Zhou, 2016).

When the time scale of feedback inhibition is much larger than that of excitation, our LIF network exhibits low-frequency oscillation burstiness (Figure 3A, left panel). This is similar to the low-frequency (about 3Hz) “spike-and-wave” EEG pattern commonly observed in absence seizure (Hughes, 2009), in which the “spike” component is associated with neuronal firing, while the “wave” is associated with hyperpolarization of neurons. In normal case, the thalamocortical (TC) cells are mainly inhibited by fast GABA_A_ currents from the thalamic reticular (RE) cells recurrently connected with them in the thalamus, and oscillates with frequency around 10 Hz. In pathological case, however, the cortical pyramidal neurons (PN) becomes hyperexcited by, say, lack of synaptic inhibition (Maheshwari and Noebels, 2014) or impaired hyperpolarization-activated current (*I*_*h*_) (Strauss et al., 2004) in PN. In this case, the strong excitatory corticothalamic feedback will evoke the IPSPs in TC cells from RE cells dominated by the slow GABA_B_ component, which lowers down the oscillation frequency to about 3 Hz (Destexhe, 2007, 2008). Therefore, absence seizure is closely related to the prolonged inhibitory time scale in the thalamus, which shares a similar mechanism as the synchronous burstiness observed in our LIF network. Our study shows that heterogeneity of cross-correlation is the main reason for the large efficacy variability in this state, which provides possible understanding to the memory deficit in children with absence seizure (Nolan et al., 2004; Henkin et al., 2005).

However, although spike shuffling methods provide important insights into the influence of spike pattern structure to synaptic plasticity, they have their own limitations. Firstly, the spike shuffling methods we use always randomize spike patterns, so that the spike patterns look more like homogeneous Poisson processes with homogeneous firing rate after being treated by each method. For example, Spike Swap (SS) is able to change the CV of stationary spike trains. But this change is always toward the direction of “randomization”: it increases (decreases) CV if that of the original spike train is smaller (larger) than 1. This may be solved using parametric spike shuffling methods. For example, spike trains can be regularized if we constrain the shuffling process using spike history effect, such as refractory period (Berry and Meister, 1998), or correlations among adjacent inter-spike intervals (Brandman and Nelson, 2002). Secondly, from Figure 5, some spike shuffling methods simultaneously strongly alter more than one pattern statistics. As a result, before we study the influence of a pattern statistics using a shuffling method, we have to first treat the spike pattern using other shuffling methods to randomize other pattern statistics thereby nullifying their influences (see Section 3.2). This limits us from understanding how a pattern statistics may influence the efficacy variability when other pattern statistics remain unchanged. From this aspect, the advantage of the strategy of our previous paper (Bi and Zhou, 2016) becomes manifested, in which the statistical features of the spike patterns can be explicitly controlled by the statistical models, which facilitates a thorough and systematic study in a large parameter range. Therefore, both the strategies in this paper and in our previous paper have their pros and cons. They complement each other, helping us to gain a thorough and convincing understanding on the problem.

During plasticity, synaptic efficacies and network dynamics interact with each other. In both of our papers, we only studied the influence of network dynamics onto the efficacy variability under STDP and synaptic homeostasis, by supposing that the neurons fire spikes according to pre-specified spike patterns, irrelevant with the synaptic weights. Although this approach saved us from the complex co-evolution of the two (Figure 3B), it does not provide a thorough understanding on the dynamics of plastic networks. Studies on the synapse-dynamics coevolution usually use the assumption that the timescale of spiking covariance is far smaller than that of plasticity, thereby focusing on the evolution of the expectations of synaptic weights (Babadi and Abbott, 2013; Ocker et al., 2015), and neglecting the trial-to-trial variabilities; or use heavy simulations (Fiete et al., 2010; Litwin-Kumar and Doiron, 2014) to get phenomenological understandings. The key difficulty here lies in the efficacy variability during the plasticity process: initial variability of synaptic weights generates different network dynamics for different trials, which then further amplifies the trial difference of network structure during plasticity. This may make the synapse-dynamics coevolution sensitive to initial conditions and noises, thereby resulting in sharply different functional performances for different individuals. How do realistic neuronal networks develop robust brain functions through the jungle of noises? We believe our work is able to help to understand this intriguing problem in future research.

## Author contributions

ZB and CZ conceived the idea and designed the research. ZB performed the research. ZB and CZ wrote the paper.

### Conflict of interest statement

The authors declare that the research was conducted in the absence of any commercial or financial relationships that could be construed as a potential conflict of interest. The reviewer CK and handling Editor declared their shared affiliation, and the handling Editor states that the process nevertheless met the standards of a fair and objective review.
