# Supplementary material for: Spike Pattern Structure Influences Synaptic Efficacy Variability under STDP and Synaptic Homeostasis. II: Spike Shuffling Methods on LIF Networks
Source: Front Comput Neurosci. 2016 Aug 9;10:83. doi: 10.3389/fncom.2016.00083 (PMC4977343; doi:10.3389/fncom.2016.00083)
Supplement: Supplementary file 2 [file DataSheet1.pdf]

Supplementary Material: Spike Pattern Structure Influences Synaptic Efficacy Variability Under STDP and Synaptic Homeostasis. II: Spike Shuffling Methods on LIF Networks

Zedong Bi* and Changsong Zhou†

*Correspondence: zedong.bi@outlook.com

†Correspondence: cszhou@hkbu.edu.hk

1 Basic properties of efficacy variability under STDP and synaptic homeostasis

Here we summarize some basic properties of efficacy variability under STDP and synaptic homeostasis, which is important for understanding this paper.

1.1 Comparing $\mathbf{E}_T(\mathbf{Var}_S(\Delta\mathbf{W}))$ with $\mathbf{Var}_T(\mathbf{E}_S(\Delta\mathbf{W}))$

In **Section 2.1** of the main text, we mentioned that the efficacy variability can be written as

$$\mathrm{Var}_{S,T}(\Delta\mathbf{W}) = \mathrm{Var}_T(\mathrm{E}_S(\Delta\mathbf{W})) + \mathrm{E}_T(\mathrm{Var}_S(\Delta\mathbf{W})), \tag{1}$$

and that in our theoretical context, $\mathrm{Var}_T(\mathrm{E}_S(\Delta\mathbf{W})) \approx 0$, so that

$$\mathrm{Var}_{S,T}(\Delta\mathbf{W}) \approx \mathrm{E}_T(\mathrm{Var}_S(\Delta\mathbf{W})). \tag{2}$$

Here, we provide numerical validation to this statement.

We implement synaptic homeostasis during the synaptic evolution under STDP, so that the mean weight of all the synapses into a neuron is kept constant (**eq.6** in the main text). Therefore, under synaptic homeostasis, $\mathrm{E}_S(\Delta\mathbf{W})$ in different trials are all the same, so $\mathrm{Var}_T(\mathrm{E}_S(\Delta\mathbf{W})) = 0$, and **eq.2** is strictly correct. To understand the situation that synaptic homeostasis is absent, in **Supplementary Figure 1**, we plot the ratio $\frac{\mathrm{Var}_T(\mathrm{E}_S(\Delta\tilde{\mathbf{W}}))}{\mathrm{E}_T(\mathrm{Var}_S(\Delta\tilde{\mathbf{W}}))}$ under the original spike patterns, in which $\Delta\tilde{\mathbf{W}}$ representing the synaptic changes only caused by STDP. We see that this ratio is very small, which suggests that we still have **eq.2** even without synaptic homeostasis.

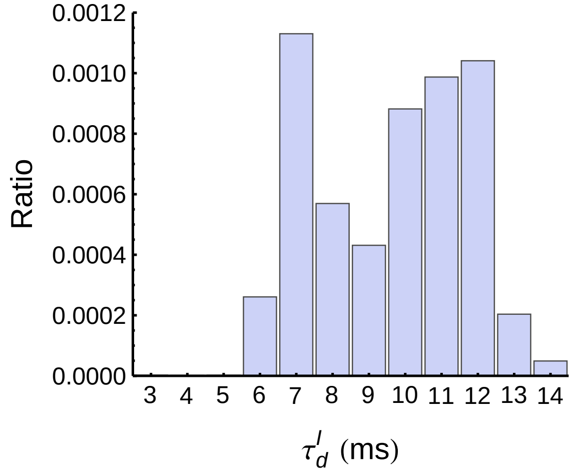

Figure 1: **Validating eq.2 in the absence of synaptic homeostasis.** Here we plot the ratio $\frac{\mathrm{Var}_T(\mathrm{E}_S(\Delta\tilde{\mathbf{W}}))}{\mathrm{E}_T(\mathrm{Var}_S(\Delta\tilde{\mathbf{W}}))}$ under the original spike patterns, in which $\Delta\tilde{\mathbf{W}}$ representing the synaptic changes only caused by STDP. When $\tau_d^I = 3\mathrm{ms}, 4\mathrm{ms}$ or $5\mathrm{ms}$, this ratio is so small that we cannot clearly see the bar representing its value in the plot. Spike patterns lasted for 41s, with STDP starting after the initial 2s of transient period. We used the synaptic strengths at the end of the STDP evolution to construct the matrix $\Delta\tilde{\mathbf{W}}$. Simulations were run for 32 trials.

1.2 Efficacy variability in converging motifs and in a general network under synaptic homeostasis

In our previous paper (Bi and Zhou, 2016), we studied the efficacy variability in converging motifs. Here we explain the relation between the efficacy variability in converging motifs and that in a general network. Using the law of total variance (Weiss, 2005), the variance of all the synaptic changes in a general plastic network can be decomposited as

$$\mathrm{Var}_{ab}(\Delta w_{ab}) = \mathrm{Var}_a(\mathrm{E}_{b\in\partial a}(\Delta w_{ab})) + \mathrm{E}_a(\mathrm{Var}_{b\in\partial a}(\Delta w_{ab})), \tag{3}$$

with Δw_{ab} being the synaptic changes from neuron b to neuron a, and ∂a being all the neurons that input to neuron a in the plastic network. Under synaptic homeostasis **eq.6** in the main text, the mean synaptic strength input to every neuron is the same, so $\mathrm{Var}_a(\mathrm{E}_{b\in\partial a}(\Delta w_{ab})) = 0$. Therefore,

$$\mathrm{Var}_{ab}(\Delta w_{ab}) = \mathrm{E}_a(\mathrm{Var}_{b\in\partial a}(\Delta w_{ab})), \tag{4}$$

which means that the efficacy variability totally comes from the efficacy variability within the converging motifs in the network (i.e., $\mathrm{Var}_{b\in\partial a}(\Delta w_{ab})$ in the second term). Therefore, converging motifs are basic units to understand the efficacy variability of a general network under synaptic homeostasis, and our previous study paved the way to understand the efficacy variability of a general network.

1.3 Some properties of DriftV in converging motifs

1.3.1 P-D imbalance and its contribution to DriftV

In a converging motif, the trial-averaged change of the ath synapse per unit time can be written as

$$v_a = \frac{\mathrm{dE}_T(\Delta w_a)}{\mathrm{d}t} = r_0 r_a \int_{-\infty}^{\infty} \mathrm{d}\tau H(\tau) C_a(-\tau_{delay} - \tau), \tag{5}$$

in which r_0 and r_a respectively being the time-averaged firing rates of the central and non-central neurons, $H(\tau)$ being the STDP time window, τ_{delay} being the axonal delay, and $C_a(\tau) \equiv \frac{\langle r_0(t) r_a(t+\tau)\rangle}{\langle r_0(t)\rangle\langle r_a(t)\rangle}$ being the unit cross-correlation between the central neuron and the ath non-central neuron. Then the DriftV of a converging motif can be quantified by

$$\mathrm{Var}_a(v_a) = r_0^2 \mathrm{Var}_a[r_a \int_{-\infty}^{\infty} \mathrm{d}\tau H(\tau) C_a(-\tau_{delay} - \tau)]. \tag{6}$$

If C_a is the same for different as, then it can be shown that

$$\mathrm{Var}_a(v_a) \propto |\mathrm{E}_a(v_a)|^2 \mathrm{Var}_a(r_a), \tag{7}$$

with $\mathrm{Var}_a(r_a)$ quantifying the heterogeneity of firing rates, and $|\mathrm{E}_a(v_a)|$ quantifying the imbalance of the strengths of the potentiation and depression process under STDP (*P-D imbalance*). This equation means that P-D imbalance is able to induce DriftV through its interaction with heterogeneity of rates.

Because of the additive nature of our synaptic homeostasis (**eq.6** in the main text), efficacy variability of converging motifs does not change after we consider the synaptic changes caused by synaptic homeostasis. However, we should notice that $\mathrm{E}_a(v_a)$ in **eq.7** means the synaptic changes only caused by STDP (without considering synaptic homeostasis). We have also pointed this out in the discussion below **eq.11** in the main text.

1.3.2 Manifesting the contributions to DriftV by P-D imbalance and heterogeneity of cross-correlations

Eq. 6 suggests that the heterogeneity of cross-correlation can influence DriftV, and our discussion above suggests that the interaction of P-D imbalance and heterogeneity of rates is another source of DriftV. These two contributions to DriftV can be explicitly split under the condition that r_a and C_a in **eq.6** are independent.

Suppose X and Y are independent random variables, then it can be proved that

$$\mathrm{Var}(XY) = \mathrm{Var}(X)\mathrm{Var}(Y) + \mathrm{Var}(X)\mathrm{E}(Y)^2 + \mathrm{Var}(Y)\mathrm{E}(X)^2.$$

Using this formula, assuming r_a and C_a are independent, **eq.6** becomes

$$\mathrm{Var}_a(v_a) = r_0^2 \mathrm{Var}_a(r_a) \cdot \{\mathrm{E}_a[\int_{-\infty}^{\infty} \mathrm{d}\tau H(\tau) C_a(-\tau_{delay} - \tau)]\}^2$$

$$+ r_0^2 \mathrm{Var}_a[\int_{-\infty}^{\infty} \mathrm{d}\tau H(\tau) C_a(-\tau_{delay} - \tau)] \cdot [\mathrm{Var}_a(r_a) + (\mathrm{E}_a(r_a))^2].$$

From **eq.5**, $\mathrm{E}_a[\int_{-\infty}^{\infty} \mathrm{d}\tau H(\tau) C_a(-\tau_{delay} - \tau)] = \frac{\mathrm{E}_a(v_a)}{\mathrm{E}_a(r_a) r_0}$, which makes the equation above becomes

$$\mathrm{Var}_a(v_a) = \frac{[\mathrm{E}_a(v_a)]^2}{[\mathrm{E}_a(r_a)]^2} \mathrm{Var}_a(r_a) + r_0^2 \mathrm{Var}_a[\int_{-\infty}^{\infty} \mathrm{d}\tau H(\tau) C_a(-\tau_{delay} - \tau)] \cdot [\mathrm{Var}_a(r_a) + (\mathrm{E}_a(r_a))^2]. \quad (8)$$

In this equation, the first term represents the contribution of the P-D imbalance (i.e. $|\mathrm{E}_a(v_a)|$) under heterogeneity of firing rates (i.e. $\mathrm{Var}_a(r_a)$), while the second term represents the contribution of heterogeneity of cross-correlations (i.e. $\mathrm{Var}_a[\int_{-\infty}^{\infty} \mathrm{d}\tau H(\tau) C_a(-\tau_{delay} - \tau)]$). When potentiation and depression balance each other (i.e. $\mathrm{E}_a(v_a) = 0$), we see that $\mathrm{Var}_a(v_a) \propto \mathrm{Var}_a[\int_{-\infty}^{\infty} \mathrm{d}\tau H(\tau) C_a(-\tau_{delay} - \tau)]$.

2 Adjusting the firing rates of the excitatory population for different τ_d^Is

As we mentioned in **Section 2.3** of the main text, in our simulations, the decay time constant τ_d^I of all the inhibitory synapses are the same in one simulation trial, but may be different for different trials. We used different c parameters for different τ_d^Is, so that the firing rates of the excitatory population are kept around 20Hz regardless of τ_d^I. In this section, we introduce the method we used to choose the c parameter under a given τ_d^I.

During our simulations, we found that the firing rate of the excitatory population tends to monotonically increase with c in a relative large parameter range. Based on this fact, given the value of τ_d^I, we used the following pseudocode to find the suitable c parameter:

1) Initialize c_{new}, c_{old} and r_{old}.

2) Set $c = c_{new}$, run the simulation for (2000+20000)s biological time, and calculate the firing rate of the excitatory population r_{new}. The initial 2000s is regarded as transient period, and is discarded when calculating the firing rate. Record c_{new} and r_{new}.

3) If $r_{old} > 20$Hz and $r_{new} > 20$Hz, then $c_{old} \leftarrow c_{new}$ and $c_{new} \leftarrow (1 - 0.004) c_{new}$; if $r_{old} < 20$Hz and $r_{new} < 20$Hz, then $c_{old} \leftarrow c_{new}$ and $c_{new} \leftarrow (1 + 0.004) c_{new}$; if only one of r_{old} and r_{new} is larger than 20Hz, then c_{new} and c_{old} are first swapped and then $c_{new} \leftarrow (c_{new} + c_{old})/2$. $r_{old} \leftarrow r_{new}$.

4) Iterate the 2nd and 3rd steps for a number of times.

After iterating the 2nd and 3rd steps above for a sufficient number of times, r_{new} starts to randomly walk around 20Hz because of the trial-to-trial variability of the calculated firing rate. From the last 15 times of the iteration, we chose the c_{new} value that corresponding to the r_{new} most close to 20Hz by eye looking, and regarded it as the c parameter for the given τ_d^I. We plot the firing rate as a function of τ_d^I under the chosen c parameters in **Supplementary Figure 2A**. The firing rates in this figure are all larger than 20Hz, which is probably due to the human bias during the eye-looking choosing. However, this does not matter, because what we care is that the firing rates of the excitatory population should be kept around *the same* value for different τ_d^Is, so that the change of efficacy variability with τ_d^I shown in **Figure 10AB** in the main text is totally due to the high-order statistics of the spike patterns. From **Supplementary Figure 2A**, we see that when τ_d^I changes, the firing rate fluctuates within 0.5% of its mean value, which is significantly smaller than the relative changes of efficacy variability with τ_d^I (**Figure 10AB** in the main text).

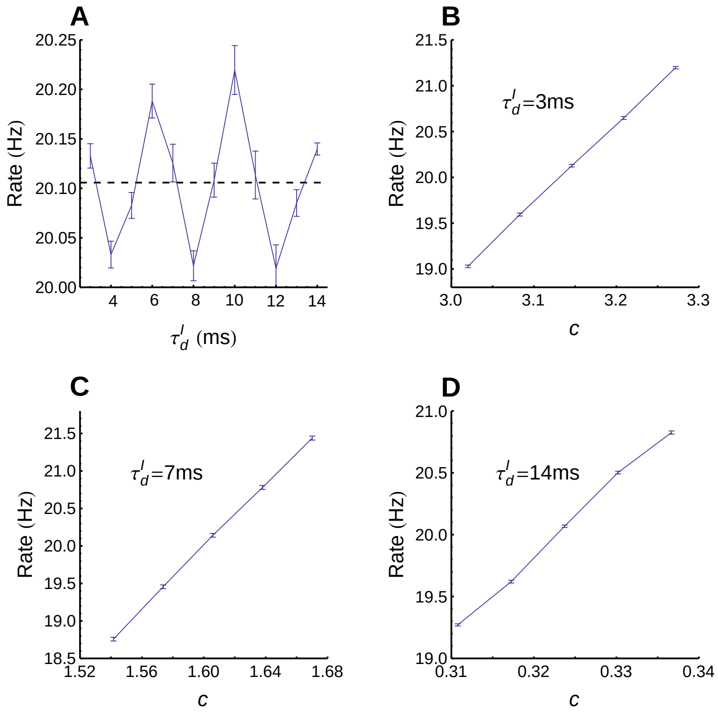

Figure 2: **Adjusting the firing rates of the excitatory population for different τ_d^Is.** (**A**) The firing rate of the excitatory population as a function of τ_d^I, when the c parameters are chosen as the values we used in our simulations (see **Section 2.3** of the main text). The dashed line represents the mean value of these firing rates. (**B-D**) The firing rate as a function of c, in the neighborhood of the c values we used in our simulations. The five points in each of these three panels respectively correspond to 90%, 95%, 100%, 105% and 110% of the c values we used in our simulations. (**A-D**) Error bars represent s.e.m. over 32 trials.

The change of firing rate with c around the chosen c parameter is exemplified in **Supplementary Figure 2B-D**.

3 Technical details of fitting c_{diffv} and c_{driftv}

As we explained in **Section 2.4** of the main text, we estimated the strengths of DiffV and DriftV by fitting the formula

$$v(t)/t = c_{diffv} + c_{driftv}/t \tag{9}$$

using the recorded evolution of efficacy variance $v(t)$ with time. In this section, we explain the technical details of this fitting.

3.1 The starting time for fitting

Under STDP, a synapse is depressed when a pre-synaptic spike gets the axonal terminal, and is potentiated when a post-synaptic spike is back-propagated to the dendritic end. Therefore, the weight of the synapse undergoes a jump process driven by the pre- and post-synaptic spike trains. When the evolution time t is large, the number of jumps

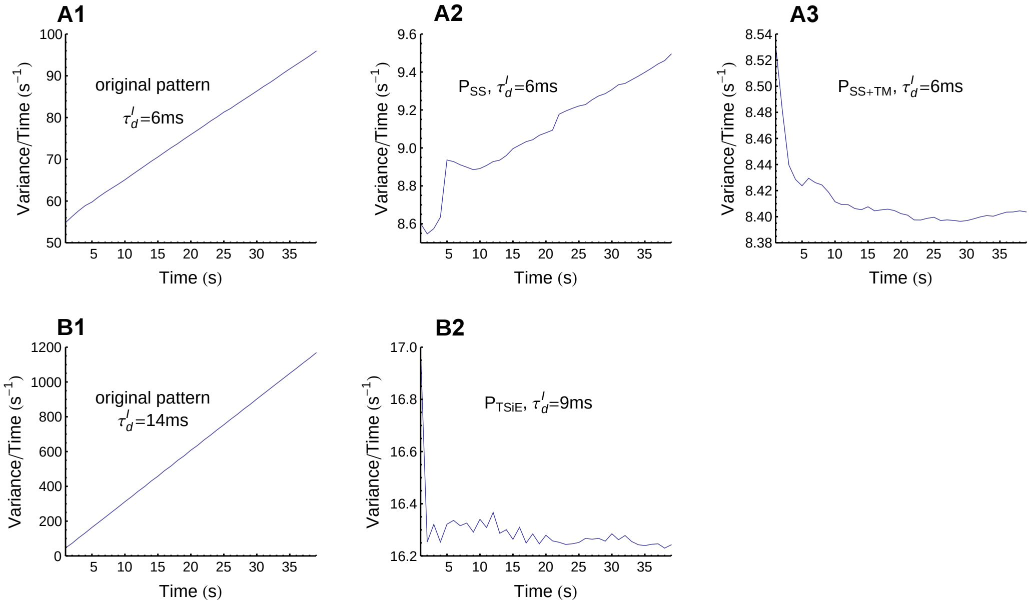

Figure 3: **The evolution of $\mathbf{E}_T(\mathbf{Var}_{(ab)}(\Delta w_{ab}))/t$ with time t under several spike patterns, with $\mathbf{Var}_{(ab)}(\Delta w_{ab})$ being the variance of synaptic changes within the excitatory population, and $\mathbf{E}_T$ representing trial average.** (**A1-A3**) The evolutions under three asynchronous patterns. DriftV is relatively strong under the original patterns (**A1**), weak under P_{SS} (**A2**), and almost zero under P_{SS+TM} (**A3**). (**B1-B2**) The evolutions under two synchronous patterns. DriftV is strong under the original patterns (**B1**), and is almost zero under P_{TSiE} (**B2**).

is also large. In this case, DiffV $\propto t$ because of the uncorrelated part of these jumps (and central limit theorem), while DriftV $\propto t^2$ because of the correlated part of these jumps. However, if t is small, the number of jumps is also small, then such relationships may not emerge. Because of this, it may not be suitable to fit the formula using the efficacy variances recorded at the very beginning of the simulations.

To estimate the suitable time t to start with, we plot the evolution of $\mathrm{E}_T(\mathrm{Var}_{(ab)}(\Delta w_{ab}))/t$ with time t under several spike patterns (**Supplementary Figure 3**), with $\mathrm{Var}_{(ab)}(\Delta w_{ab})$ being the variance of synaptic changes within the excitatory population, and E_T representing trial average. According to **eq.9**, we should expect that $\mathrm{E}_T(\mathrm{Var}_{(ab)}(\Delta w_{ab}))/t$ is linear with time t. However, we found that this linearity is only significant for sufficiently large t, especially for spike pattens under which DriftV is small (see **Supplementary Figure 3A2,A3,B2**). After considering this fact, we chose $t = 15$s as the time when we started to use the recorded efficacy variances for our fitting.

3.2 The goodness of fit

In our simulations, we recorded the efficacy variance at a given time for 32 trials. Because of this, we use lack-of-fit F-statistic (Walpole et al, 2012) to assess the goodness of fit.

F-statistic is defined by

$$F = \frac{N_{trial} \sum_{t=t_1}^{t_{N_{time}}} (\overline{v(t)} - \widehat{v(t)})^2/(N_{time} - 2)}{\sum_{i=1}^{N_{trial}} \sum_{t=t_1}^{t_{N_{time}}} (v_i(t) - \widehat{v(t)})^2/(N_{trial}N_{time} - N_{time})},$$

with $v_i(t)$ being the variance of synaptic changes at time t in the ith simulation trial, $\overline{v(t)}$ being the average variance over all the trials, $\widehat{v(t)}$ being the variance predicted by the model, $N_{trial} = 32$ being the number of trials, and $N_{time} = 25$ being the number of time points at which we recorded the variances. F-statistic quantifies the fit error per degree of freedom caused by the lack of fit of the model relative to the experimental error (here, the trial-to-trial variability). Small F-statistic means that the lack of fit of the model is weaker than the experimental error, which suggests that the proposed model well explains the experimental data; while large F-statistic has the opposite meaning.

In **Supplementary Figure 4A1, B1, C1**, we exemplify the F-statistics under the original spike patterns, P_{SS} in asynchronous states and P_{TSiE} in synchronous states; we see that most of the F-statistics are very small. For the highest F-statistics under each type of patterns, we also calculate the p-values for the null hypothesis that "there is no lack of linear fit" (Walpole et al, 2012), and get high p-values in all cases. These facts suggest that our model fits the data quite well. We also draw the scatter plot and the fitting line for the lowest p-value cases (i.e., highest F-statistic cases) under each type of patterns in **Supplementary Figure 4A2-A3, B2, C2**, exemplying the worst fittings.

4 AT$_{\text{WithinEvent}}$ in P_{TSiE}, $P_{TSiE+SSiE}$ and $P_{TSiE+NRC}$

In the main text, we use $CV_{WithinEvent}$ to quantify AT$_{\text{WithinEvent}}$, which is the mean CV value of the pieces of spike trains that contain more than 2 spikes in synchronous events. However, if a spike train contains only a small number of spikes, its CV may also depends on the number of spikes it contains. For example, the CV of a long Poisson spike train with stationary firing rate is typically 1, but if we calculate the CV of a short piece of this spike train which contains, say, 3 or 4 or 5 spikes, we will find that its CV is typically smaller than 1. To get more understanding on the AT$_{\text{WithinEvent}}$ in P_{TSiE}, $P_{TSiE+SSiE}$ and $P_{TSiE+NRC}$, we will discuss CV as a function of the spike number n in a synchronous event under these three patterns in this section.

We found that in all these three patterns, $CV(n)$ tends to increase with n (**Supplementary Figure 5, upper panels**). CV in P_{TSiE} is smaller than that in $P_{TSiE+SSiE}$ for all n values, which reflects the increase of the burstiness of AT$_{\text{WithinEvent}}$ caused by SSiE. We also found that distribution of the spike number n is narrow under P_{TSiE} and $P_{TSiE+SSiE}$, and gets broadened in $P_{TSiE+NRC}$ (**Supplementary Figure 5, middle and lower panels**). However, for the n values whose probability is nonzero under $P_{TSiE+SSiE}$, its $CV(n)$ is almost the same as the $CV(n)$ under $P_{TSiE+NRC}$ (**Supplementary Figure 5, upper panels**). This suggests that the spike times under $P_{TSiE+SSiE}$ are randomized to the same extent as those under $P_{TSiE+NRC}$.

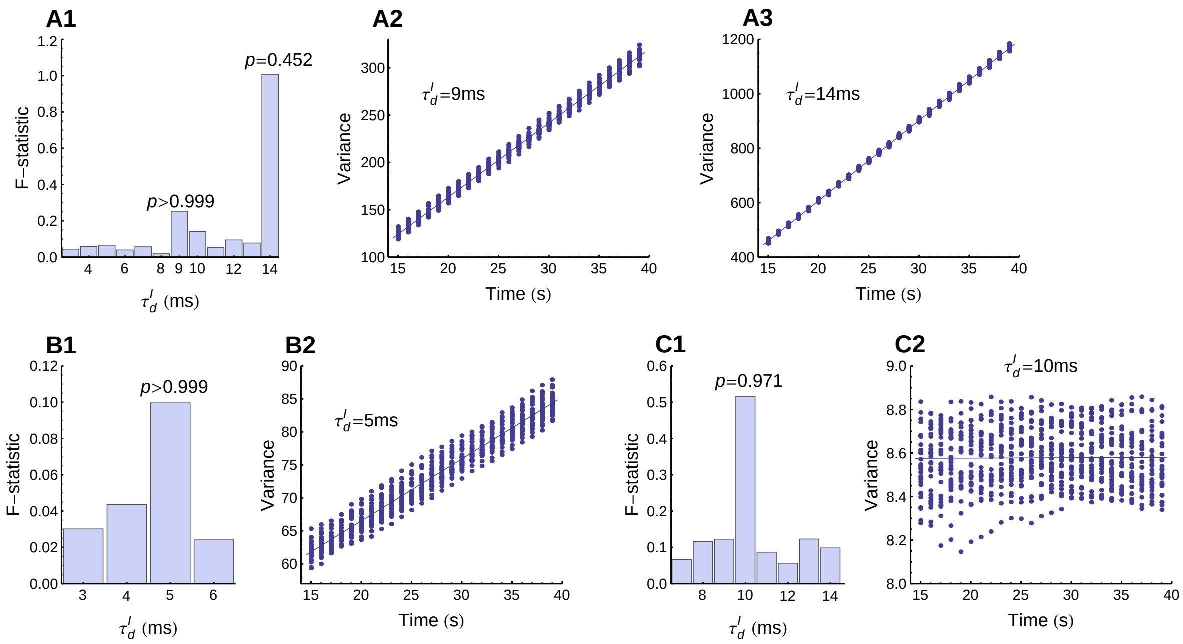

Figure 4: **The goodness of fit.** (**A1-A3**) Investigating the goodness of fit under the original spike patterns. In **A1**, we show the F-statistics for different τ_d^Is, and also the p-values for the null hypothesis that "there is no lack of linear fit" for the two τ_d^Is with highest F-statistics. In **A2** and **A3**, we draw the scatter plots and the fitting lines for these two τ_d^Is to exemplify the worse fittings. (**B1-B2**) The F-statitics, p-value, scatter plot and fitting line under P_{SS} in asynchronous states. (**C1-C2**) The F-statistics, p-value, scatter plot and fitting line under P_{TSiE} in synchronous states.

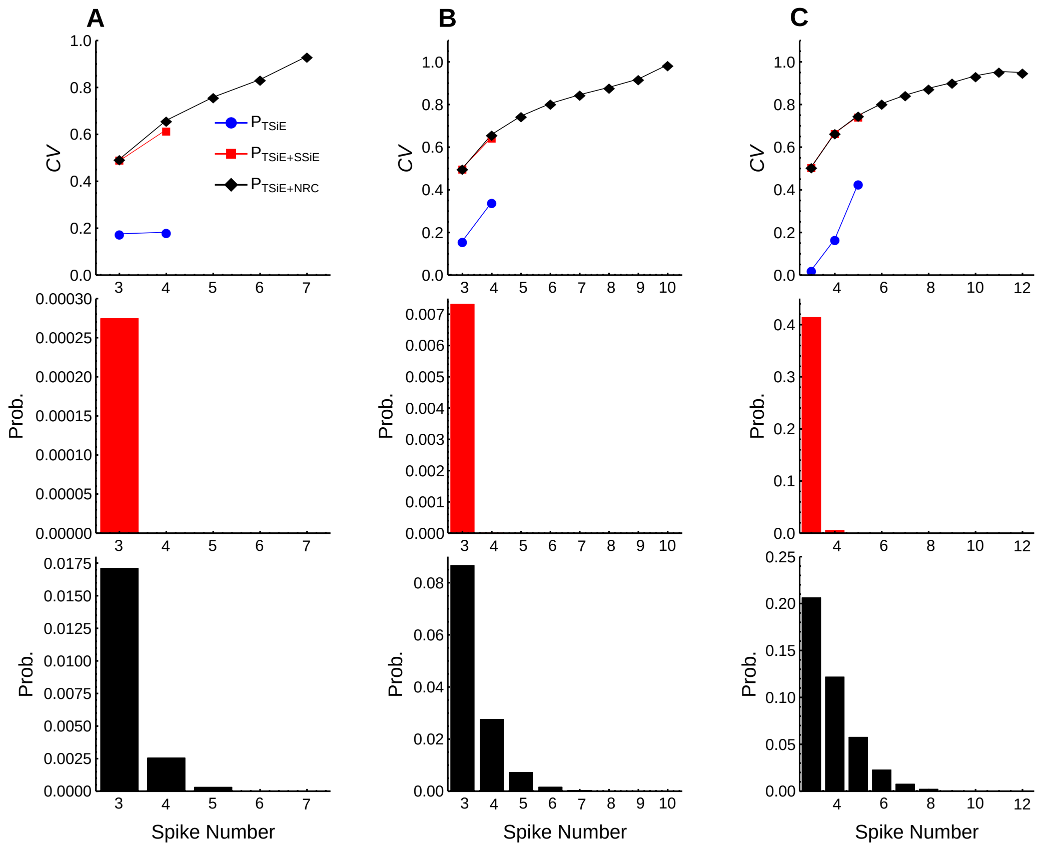

Figure 5: **Understanding $\mathbf{AT_{WithinEvent}}$ in P_{TSiE}, $P_{TSiE+SSiE}$ and $P_{TSiE+NRC}$.** (**A**) Upper: The expectation of the CV of a piece of spike train in a synchronous event as a function of the spike number n that the piece of spike train contains, when $\tau_d^I = 7$ms. The CV is only defined when $n \geq 3$, and the probability that $n \geq 5$ in P_{TSiE} and $P_{TSiE+SSiE}$ is zero, so $CV(n)$ is only meaningful when $n = 3, 4$ in P_{TSiE} and $P_{TSiE+SSiE}$. Note that for the meaningful n values, the CV in $P_{TSiE+SSiE}$ is very close to that in $P_{TSiE+NRC}$. Middle: The probability distribution of n under $P_{TSiE+SSiE}$. We only shows the values when $n \geq 3$. And this distribution under $P_{TSiE+SSiE}$ is exactly the same with that under P_{TSiE}. Lower: The distribution of n under $P_{TSiE+NRC}$. (**B**) The same as **A**, except that $\tau_d^I = 11$ms. (**C**) The same as **A**, except that $\tau_d^I = 14$ms. Simulation details are the same as **Figure 9** in the main text. Error bars are now shown for simplicity.

5 Comparing the results of this paper with the results of our previous paper

In this paper, we investigate the influence of spike pattern structure onto efficacy variability by implementing spike shuffling methods onto spike patterns self-organized by a LIF networks; in our previous paper (Bi and Zhou, 2016), we investigated a similar problem using spike generating models on converging motifs. In this section, we briefly review the results of our previous paper, and compare them with the results of this paper. We find that the results of this paper provide further validation to some of the results of our previous paper.

For the convenience of the following discussions, we call the neuron that receives from many neurons in a converging motif to be the *central neuron*, and call the other neurons *non-central neurons*.

5.1 DiffV

5.1.1 Auto-correlation structure in stationary spike trains

Results of the previous paper:

If spike trains of all the neurons (including the non-central and central neurons) in a converging motif are stationary (i.e., firing rates do not change with time) with equal firing rate, then DiffV is smallest when CV is within the range $0.3 \sim 0.7$, and gets large either when the spike trains get burstier or more regular. If different non-central neurons have different firing rates, then DiffV tends to monotonically increases with CV.

Results of this paper:

For asynchronous spike patterns, we compared P_{TM} with P_{TM+SS} (Term 3 in **Section 3.3** of the main text). We found that $CV > 1$ in P_{TM}, but SS reduces CV to almost 1. At the same time, c_{diffv} is larger under P_{TM} than under P_{TM+SS} , which suggests that the burstiness of stationary spike trains increases DiffV. This is consistent with our previous result in the $CV > 1$ case.

5.1.2 Heterogeneity of rates in stationary spike trains

Results of the previous paper:

For stationary spike trains, introducing heterogeneity of rates to non-central neurons does not strongly influence DiffV when the spike trains are bursty, but discounts the increase of DiffV with the decrease of CV under strong regularity.

Results of this paper:

For asynchronous spike patterns, we compared P_{TM+SS} with P_{TM+NRC} (Term 2 in **Section 3.3** of the main text). We found that $CV \approx 1$ in both these two patterns, and that c_{diffv} is almost the same under these two patterns, which suggests that heterogeneity of rates does not influence DiffV when CV is around 1. This is consistent with our previous result in the burstiness case.

5.1.3 Synchronous firing

Results of the previous paper:

Synchronous firing usually increases DiffV, except for spike-to-spike synchrony and good temporal precision.

Results of this paper:

For asynchronous spike patterns, we compared P_{SS} with P_{SS+TM} to investigate the effect of synchronous firing (Term 1 in **Section 3.3** of the main text). Here, for asynchronous spike patterns, synchronous firing means the fluctuation of population firing rate with time. We found that c_{diffv} is larger under P_{SS} than under P_{SS+TM}, which suggests that synchronous firing facilitates DiffV. This is consistent with our previous result.

For synchronous spike patterns, we compared P_{SSiE} with $P_{SSiE+STR}$, and found that c_{diffv} is larger under P_{SSiE} than under $P_{SSiE+STR}$ (Term 1 in **Section 3.4** of the main text). In our spike patterns, there is no spike-to-spike synchrony, so this result is also consistent with our previous result.

5.1.4 Auto-correlation structure under synchronous firing

Results of the previous paper:

Under synchronous firing, auto-correlation structure may have a number of types, such as $\text{AT}_{\text{WithinEvent}}$, $\text{AT}_{\text{SpikeNum}}$ and $\text{AT}_{\text{events}}$ (see **Figure 4** in the main text). Burstier spike trains within synchronous events (for $\text{AT}_{\text{WithinEvent}}$), broader distribution of the spike numbers a neuron fires in different synchronous events (for $\text{AT}_{\text{SpikeNum}}$), and burstier occurrences of synchronous events (for $\text{AT}_{\text{events}}$) tend to increase DiffV.

Results of this paper:

1) To investigate the effect of $\text{AT}_{\text{WithinEvent}}$, we compared P_{TSiE} with $P_{TSiE+SSiE}$ (Term 3 in **Section 3.4** of the main text). We found that pieces of spike trains within synchrous events are burstier under $P_{TSiE+SSiE}$ than under P_{TSiE}, and c_{diffv} is also larger under $P_{TSiE+SSiE}$ than under P_{TSiE}. This is consistent with our previous result.

2) To investigate the effect of $\text{AT}_{\text{SpikeNum}}$, we compared $P_{TSiE+SSiE}$ with $P_{TSiE+NRC}$ (Term 4 in **Section 3.4** of the main text). We found that the spike number distribution is broader under $P_{TSiE+NRC}$ than under $P_{TSiE+SSiE}$, and c_{diffv} is also larger under $P_{TSiE+NRC}$ than under $P_{TSiE+SSiE}$. This is consistent with our previous result.

3) To investigate the effect of $\text{AT}_{\text{events}}$, we compared P_{SSiE} with $P_{SSiE+ETS}$ (Term 5 in **Section 3.4** of the main text). We found that the occurrence of synchronous events is burstier under $P_{SSiE+ETS}$ than under P_{SSiE}, but c_{diffv} is almost the same under these two patterns. However, we argue here that this may be because that the contribution to DiffV from the interactions between spikes in different synchronous events are small due to the far separation between synchronous events and the decaying STDP time window.

To study the effect of $\text{AT}_{\text{events}}$ onto DiffV in more details, we compare c_{diffv} under $P_{TSiE+SSiE}$ with that under $P_{TSiE+SSiE+ETS}$. Here we first implement TSiE to homogenize firing rate, so that DriftV $= 0$ in both these patterns. In this case, the efficacy variability is totally caused by DiffV, so that the estimated value of c_{diffv} will become more precise. We find that c_{diffv} under $P_{TSiE+SSiE+ETS}$ is larger than that under $P_{TSiE+SSiE}$ when $\tau_d^I \leq 10$ms, but there is hardly any difference when $\tau_d^I \geq 11$ms (**Supplementary Figure 6A**). This is possibly because that when τ_d^I is large, the frequency of the occurrence of synchronous events becomes lower, which separates two adjacent synchronous events farther away in time (see **Figure 3A** in the main text). This weakens the STDP interactions between the spikes in adjacent synchronous events, so that changing $\text{AT}_{\text{events}}$ does not significantly influences the synaptic changes. To validate this idea, we enlarge the STDP time scale τ_{STDP} (**eq. 5** in the main text) from the default value 20ms to 50ms, to increase the interactions between spikes in adjacent synchronous events. Indeed, we found that in this case, c_{diffv} under $P_{TSiE+SSiE+ETS}$ becomes significantly larger than that under $P_{TSiE+SSiE}$ (**Supplementary Figure 6A, inset**). This validates the result in our previous paper (Bi and Zhou, 2016) that the burstiness of the occurrence of synchronous events increases DiffV.

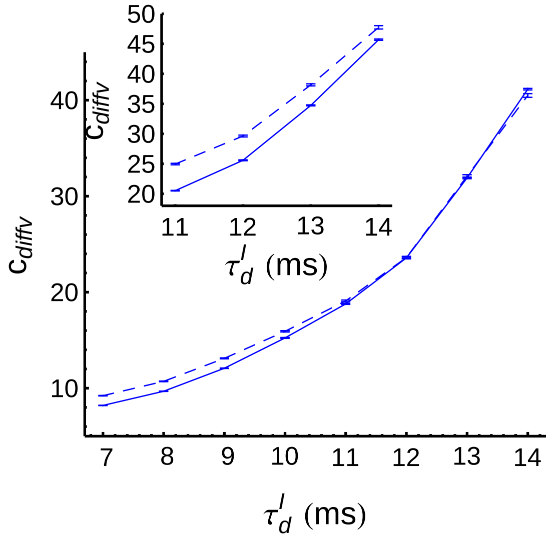

Figure 6: **Understanding the effect of $\mathbf{AT_{events}}$ to DiffV**. c_{diffv} as a function of τ_d^I under $P_{TSiE+SSiE}$ (solid line) and under $P_{TSiE+SSiE+ETS}$ (dashed line) when τ_{STDP} takes its default value 20ms. Inset: when $\tau_{STDP} = 50$ms. $A_p = A_d = 1$, error bars represent s.e.m. Simulation details are the same as those in **Figure 9** in the main text.

5.2 DriftV:

5.2.1 Auto-correlation structure in stationary spike trains

Results of the previous paper:

Auto-correlation structure in stationary spike trains does not influence DriftV.

Results of this paper:

For asynchronous spike patterns, we compared P_{TM} with P_{TM+SS} (Term 3 in **Section 3.3** of the main text). We found that $CV > 1$ in P_{TM}, but SS reduces CV to almost 1. However, we found that c_{driftv} is almost the same under these two patterns, which suggests that the burstiness of stationary spike trains does not change DriftV. This is consistent with our previous result.

5.2.2 Heterogeneity of rates

Results of the previous paper:

Under the absence of heterogeneity of cross-correlations, the interaction of heterogeneity of rates and P-D imbalance induces DriftV.

Results of this paper:

For asynchronous spike patterns, we compared P_{TM+SS} with P_{TM+NRC} (Term 4 in **Section 3.3** of the main text). We found that c_{driftv} is always near to zero under P_{TM+NRC} (whose firing rates are homogeneous), and is positively correlated with the strength of P-D imbalance under P_{TM+SS} (whose firing rates are heterogeneous). This shows that DriftV can be induced by the interaction of heterogeneity of rates and induces DriftV, which is consistent with our previous result.

For synchronous spike patterns, we compared P_{SSiE} with $P_{SSiE+TSiE}$ (Term 2 in **Section 3.4** of the main

text). We found that c_{driftv} is always near to zero under $P_{SSiE+TSiE}$ (whose firing rates are homogeneous), and is positively correlated with the strength of P-D imbalance under P_{SSiE} (whose firing rates are heterogeneous). This also shows that DriftV can be induced by the interaction of heterogeneity of rates and induces DriftV, which is again consistent with our previous results.

5.2.3 Synchronous firing

Results of the previous paper:

Synchronous firing can influence P-D imbalance $|\mathrm{E}_a(v_a)|$, thereby influencing DriftV under heterogeneity of rates.

Results of this paper:

For asynchronous spike patterns, we compare P_{SS} with P_{SS+TM} (Term 1 in **Section 3.3** of the main text). We found that when $A_p = A_d$, both c_{driftv} and $|\mathrm{E}_a(v_a)|$ are larger than zero in P_{SS} but almost zero in P_{SS+TM}, which suggests that synchronous firing influences DriftV under heterogeneity of rates by changing P-D imbalance. This is consistent with our previous result.

For asynchronous spike patterns, we compare P_{SSiE} with $P_{SSiE+STR}$ (Term 1 in **Section 3.4** of the main text). We found that when $A_p = A_d$, both c_{driftv} and $|\mathrm{E}_a(v_a)|$ are larger than zero in P_{SSiE} but almost zero in $P_{SSiE+STR}$, which also suggests that synchronous firing influences DriftV under heterogeneity of rates by changing P-D imbalance. This is again consistent with our previous result.

5.2.4 Auto-correlation structure under synchronous firing

Results of the previous paper:

As for the three types of auto-correlation structure under synchronous firing (**Figure 4** in the main text), $\mathrm{AT_{WithinEvent}}$ and $\mathrm{AT_{SpikeNum}}$ do not change P-D imbalance, thereby nor DriftV under heterogeneity of rates; $\mathrm{AT_{events}}$ changes P-D imbalance, thereby changing DriftV under heterogeneity of rates. The influence of $\mathrm{AT_{events}}$ onto P-D imbalance is complicated, but if $\tau_{delay} \ll \tau_{STDP}$ and synchronous events do not overlap with each other, $\mathrm{E}_a(v_a)$ will increase (or decrease) with the burstiness of the occurrence of synchronous events if $A_p > A_d$ (or $A_p < A_d$).

Results of this paper:

1) To investigate the effect of $\mathrm{AT_{WithinEvent}}$, we compared P_{TSiE} with $P_{TSiE+SSiE}$ (Term 3 in **Section 3.4** of the main text). We found that P-D imbalance is almost the same in these two patterns, which suggests that $\mathrm{AT_{WithinEvent}}$ does not change DriftV under heterogeneity of rates. This is consistent with our previous result.

2) To investigate the effect of $\mathrm{AT_{SpikeNum}}$, we compared $P_{TSiE+SSiE}$ with $P_{TSiE+NRC}$ (Term 4 in **Section 3.4** of the main text). We found that P-D imbalance is almost the same in these two patterns, which suggests that $\mathrm{AT_{SpikeNum}}$ does not change DriftV under heterogeneity of rates. This is consistent with our previous result.

3) To investigate the effect of $\mathrm{AT_{events}}$, we compared P_{SSiE} with $P_{SSiE+ETS}$ (Term 5 in **Section 3.4** of the main text). We carefully designed the method of ETS so that synchronous events do not overlap with each other in the spike patterns treated by ETS. We found that $P_{SSiE+ETS}$ has larger CV_{events} value than P_{SSiE}, and $\mathrm{E}_a(v_a)$ in $P_{SSiE+ETS}$ is larger (or smaller) than that in P_{SSiE}. This suggests that $\mathrm{E}_a(v_a)$ will increase (or decrease) with CV_{events} if $A_p > A_d$ (or $A_p < A_d$), which is consistent with our previous result.

5.2.5 Heterogeneity of cross-correlations

Results of the previous paper:

Heterogeneity of cross-correlations induces DriftV together with heterogeneity of rates (**eq.6**).

Results of this paper:

For asynchronous spike patterns, we compared P_{STR} and P_{STR+TM} (Term 2 in **Section 3.3** of the main text). In this two patterns, potentiation and depression are almost balanced with each other, and P_{STR} has larger heterogeneity of cross-correlations than P_{STR+TM}. We found that c_{driftv} is larger in P_{STR} than in P_{STR+TM}. This is consistent with the result derived from **eq.8**.

For synchronous spike patterns, we compared P_{WTS} and P_{SSiE} (**Section 3.5** of the main text). We found that DriftV in P_{SSiE} decreases with τ_d^I, while both DriftV and heterogeneity of cross-correlations increase with τ_d^I, which suggests that heterogeneity of cross-correlations is the main reason of DriftV when the network works in synchronous bursting states.

6 Several other topics

6.1 The change of efficacy variability with τ_d^I in the original spike patterns

The change of efficacy variability with τ_d^I in the original spike patterns is shown in **Figure 7**.

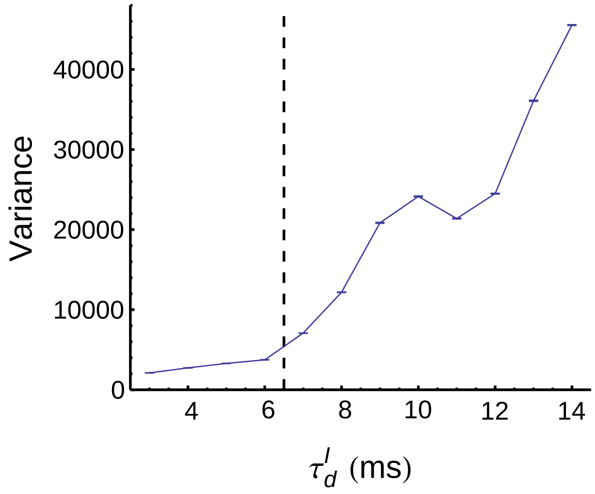

Figure 7: **Efficacy variability as a function of τ_d^I in the original spike patterns.** The dashed vertical line indicates the transition between asynchronous state and synchronous state. We used the synaptic strengths at the end of the STDP evolution to calculate the efficacy variability. Simulation details are the same as those in **Figure 10** in the main text.

6.2 The influence of STR onto CV and heterogeneity of cross-correlations in asynchronous states

We compare the CV and index of heterogeneity of cross-correlations (HCC) under P_{WTS} and P_{STR} in **Supplementary Figure 8**. We see that STR slightly reduces both of these two quantities.

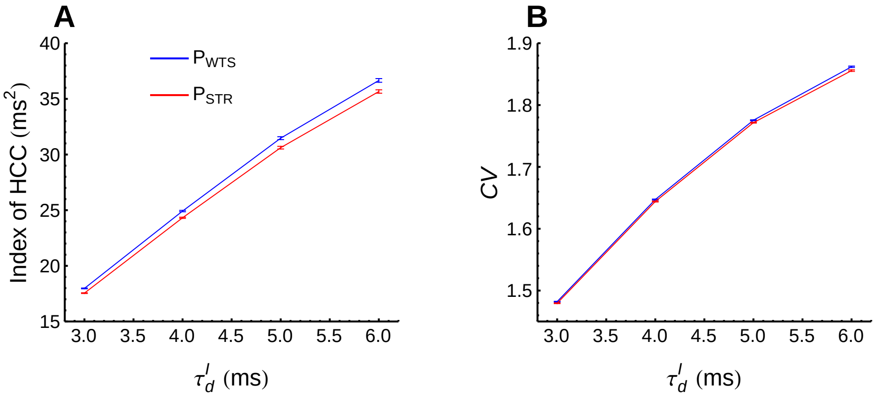

Figure 8: The index of HCC (**A**) and CV (**B**) under P_{WTS} and P_{STR} for asynchronous states.

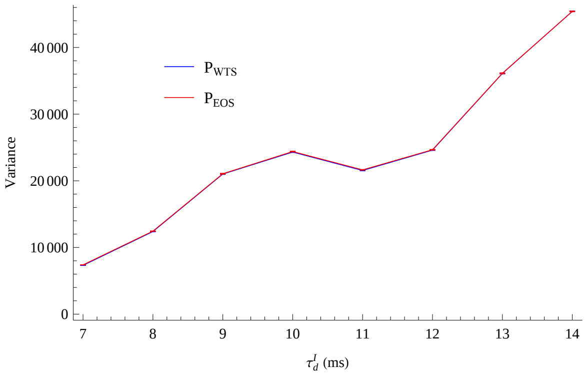

Figure 9: The efficacy variability caused by the spike patterns after WTS (blue) and after EOS (red) for synchronous states.

6.3 The influence of EOS onto the efficacy variability

EOS hardly changes the efficacy variability in synchronous spike patterns (**Supplementary Figure 9**), which suggests that the dependences (on, say, spike times or spike numbers) between the pieces of spike trains in adjacent synchronous events do not have significant influence onto the efficacy variability our model.

References

Bi, Z. and Zhou, C. (2016). Spike pattern structure influences synaptic efficacy variability under STDP and synaptic homeostasis. I: spike generating models on dendritic motifs. *Front. Comput. Neurosci.* 10, 14

Weiss, N. A. (2005). A Course in Probability (Boston: Addison-Wesley)

Walpole, R. E. and Myers, R. H. and Myers, S. L. and Ye, K. (2012). Probability & Statistics for Engineers & Scientists, 9th ed. (Boston: Pearson)
